# Disrupted macrophage metabolic reprogramming in aged soleus muscle during early recovery following disuse atrophy

**DOI:** 10.1111/acel.13448

**Published:** 2021-08-08

**Authors:** Dennis K. Fix, H. Atakan Ekiz, Jonathan J. Petrocelli, Alec M. Mckenzie, Ziad S. Mahmassani, Ryan M. O'Connell, Micah J. Drummond

**Affiliations:** ^1^ Molecular Medicine Program Department of Integrative Physiology and Nutrition Department of Physical Therapy and Athletic Training University of Utah Salt Lake City Utah USA; ^2^ Department of Pathology Division of Microbiology and Immunology University of Utah Salt Lake City Utah USA

**Keywords:** glycolysis, inflammation, metabolomics, scRNASeq, single cell transcriptomics

## Abstract

Aged skeletal muscle is characterized by poor muscle recovery following disuse coinciding with an impaired muscle pro‐inflammatory macrophage response. Macrophage inflammatory status is regulated by its metabolic state, but little is understood of macrophage metabolism and its relation to macrophage inflammation in the context of muscle recovery and aging. Therefore, the purpose of this study was to thoroughly characterize macrophage metabolism and inflammation in aged muscle during early recovery following disuse atrophy using single cell transcriptomics and functional assays. Young (4–5 months) and old (20–22 months) male C57BL/6 mice underwent 14 days of hindlimb unloading followed by 4 days of ambulatory recovery. CD45+ cells were isolated from solei muscles and analyzed using 10x Genomics single cell RNA sequencing. We found that aged pro‐inflammatory macrophage clusters were characterized with an impaired inflammatory and glycolytic transcriptome, and this dysregulation was accompanied by a suppression of HIF‐1α and its immediate downstream target, Glut1. As a follow‐up, bone marrow‐derived macrophages were isolated from a separate cohort of young and old mice at 4‐d recovery and were polarized to a pro‐inflammatory phenotype and used for glycolysis stress test, phagocytosis activity assay, and targeted GC‐MS metabolomics. Aged bone marrow‐derived pro‐inflammatory macrophages were characterized with impaired glycolysis and phagocytosis function, decreased succinate and an accumulation of glycolytic metabolic intermediates overall supporting reduced glycolytic flux and macrophage function. Our results indicate that the metabolic reprograming and function of aged skeletal muscle pro‐inflammatory macrophages are dysfunctional during early recovery from disuse atrophy possibly attributing to attenuated regrowth.

## Graphical abstract

Summary of metabolic dysfunction in aged pro‐inflammatory macrophages after 4 days of recovery from disuse atrophy. Figure depicts aged skeletal muscle macrophages during the early recovery phase following disuse atrophy (4 days of reloading). Aged pro‐inflammatory macrophages during recovery are present with impaired inflammation and glycolytic metabolism and accompanied by lower HIF‐1α. The lack of HIF‐1α transcription in the single cell sequencing may be regulated by the presence of succinate which typical increases in normal (young) pro‐inflammatory macrophages.

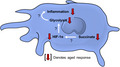

## 1 INTRODUCTION

Impaired muscle regrowth following a period of disuse in aged skeletal muscle is a widely recognized and studied phenomenon (Wang et al., 2019). Aged skeletal muscle often fails to achieve the muscle quality that it had prior to disuse atrophy (Reidy, McKenzie, et al., 2019). Failed muscle recovery following disuse events may contribute to age‐related muscle and functional decline (i.e., sarcopenia; Kanazawa et al., 2017). Unfortunately, there are numerous gaps in the understanding of the cellular and molecular processes during the regrowth phase of aged skeletal muscle.

An important contributor to skeletal muscle regrowth is a properly coordinated immune response triggered by extracellular and intracellular cues that recruit circulating immune cells to the area of damage (Duchesne et al., 2017). Recent work has highlighted the role of macrophages as an essential component of skeletal muscle regrowth following disuse atrophy (Tidball & Wehling‐Henricks, 2007) with much less work in aging (Reidy, Dupont‐Versteegden, & Drummond, 2019). Macrophages that are recruited to the cite of damage during the early phase of recovery are referred to as pro‐inflammatory and exhibit inflammatory functional properties secreting chemokines and cytokines such as interleukin‐1β (IL‐1β), tumor necrosis factor α (TNF‐α), and chemokine ligand 3 (CCL3; Viola et al., 2019). Conversely, anti‐inflammatory macrophages polarize upon completion of the initial inflammatory response and are involved in tissue repair and promote collagen synthesis (Wang et al., 2015). Though the nomenclature of pro‐ and anti‐inflammatory define two distinct populations of macrophages, it is important to denote that that macrophages exist under a dynamic spectrum of activation states (Chazaud, 2020). Studies that have investigated aged muscle damage and regeneration often reveal a blunted or delayed inflammatory profile (Reidy, McKenzie, et al., 2019; Wang et al., 2019). During the aging process, skeletal muscle becomes mores fibrotic and recent evidence has shown this is concomitant with dominance in resident macrophages presenting with an anti‐inflammatory phenotype (Cui et al., 2019). Furthermore, our laboratory group has recently demonstrated that pro‐inflammatory macrophage accumulation is reduced in the skeletal muscle of aged mice during recovery from disuse atrophy which corresponded with impaired muscle recovery (Reidy, Dupont‐Versteegden, & Drummond, 2019; Reidy, McKenzie, et al., 2019). Therefore, macrophage inflammatory status may be a source of dysfunction in aged muscle during recovery from disuse.

It is evident that macrophage polarization (i.e., inflammatory profile) and function are dependent on its metabolic state (Freemerman et al., 2014). Anti‐inflammatory macrophages rely heavily on oxidative phosphorylation to maintain energy homeostasis (Viola et al., 2019). On the other hand, pro‐inflammatory macrophages exhibit a glycolytic phenotype while also utilizing the pentose phosphate pathway to drive lipid biosynthesis and promote cytokine secretion (Viola et al., 2019). Glycolytic metabolism of pro‐inflammatory macrophages is tightly linked to their functional properties such as phagocytosis activity (Arnold et al., 2007). Furthermore, pro‐inflammatory macrophages are characterized with a truncated TCA cycle that leads to an intracellular accumulation of the intermediates including succinate and isocitrate (Viola et al., 2019). These intermediates are believed to regulate the inflammatory signaling program that commonly characterizes the inflammatory status of macrophages (Jing et al., 2020). For example, in cultured bone marrow‐derived macrophages, inhibiting glycolysis suppressed the LPS‐induced upregulation of IL‐1β and TNF‐α and prevented the accumulation of succinate (Cramer et al., 2003). Little has been done to examine these cellular processes in the context of aging and recovery following disuse in aged skeletal muscle.

In this study, we utilized fluorescent activated cell sorting of CD45+ (leukocyte antigen cell surface marker) and subsequent single cell RNA sequencing to identify and characterize the metabolic and inflammatory transcriptome of pro‐inflammatory macrophages that commonly infiltrate muscle during early recovery from disuse atrophy. This novel and powerful approach, which also includes a cell identification method that we developed (Ekiz et al., 2020), enabled us to distinguish between specific leukocytes and macrophages (myeloid‐derived) cell types and assess their function and metabolic states based upon gene expression signatures from thousands of individual cells using a single assay. Such results would not be obtainable using other conventional methods. Therefore, the purpose of this study was to examine inflammatory macrophage metabolism in aged skeletal muscle during an early time point of regrowth following disuse atrophy. We hypothesized that aging would impair the metabolic phenotype and function of pro‐inflammatory macrophages during early recovery following disuse atrophy.

## 2 METHODS

### 2.1 Animals

Male C57BL/6 young adult (4–5 months, University of Utah Genomics Core) and old (20–22 months, National Institute on Aging) mice were used in this study. Animals were housed with ad libitum access to food and water and maintained on a 12:12‐h light–dark cycle. All experimental procedures were conducted in accordance with the guidelines set by The University of Utah Institutional Animal Care and Use Committee.

### 2.2 Hindlimb unloading and reloading

Young and old mice were divided into three experimental groups: ambulatory controls (CON), 14 days of hindlimb unloading (HU), and HU followed by 4 days of reloading (RL4). Approximately 8–10 young and 8–20 old mice were assessed at CON and HU time points, respectively, whereas ~10–12 young and old mice per group were assessed during reloading. Animals assigned to CON were able to freely ambulate in their cage (2–3 animals/cage) and had ad libitum access to food (standard chow) and water during the experimental periods. For the HU and RL groups, animals underwent hindlimb suspension (2 animals/cage) using a modified unloading method based on the traditional Morey‐Holton design for studying disuse atrophy in rodents as we have done before (Reidy, McKenzie, et al., 2019). Body weight and food intake were monitored every other day to ensure that mice were not experiencing excessive weight loss due to malnutrition or dehydration. Following *day 14* of HU, HU animals were fasted for 5 h and then euthanized for soleus muscle weight assessment and single cell RNA sequencing processing (below), while the RL animals were removed from the suspension apparatus and then housed in individual cages for 4 days with tissue samples processed afterwards in the same way as detailed above. The soleus muscle was selected for analysis due its robust response to atrophy and elevated macrophage content compared to a larger, less oxidative muscle like the gastrocnemius (Reidy, Dupont‐Versteegden, & Drummond, 2019; Reidy, McKenzie, et al., 2019). Previous literature from us and others has demonstrated that 4 days is an optimal time point to capture macrophage infiltration during early recovery (Reidy, McKenzie, et al., 2019; Tidball & Wehling‐Henricks, 2007). For cell culture experiments (below), bone marrow‐derived macrophages were isolated from a second cohort of young (*N* = 4–6/time point) and old (*N* = 4–6/time point) ambulatory controls and RL4 mouse groups as described above.

### 2.3 FACS and CD45+ 10× genomics single cell RNA sequencing

For single cell RNA sequencing, soleus muscles from each time point and age group were carefully dissected and digested using liberase and DNAse for 5 min at 37°C. To acquire enough cells for scRNAseq, solei from both hindlimbs of each mouse and from an appropriate number of mice were pooled together for each treatment time point and by age such that there was a total of 6 samples (Young: Ambulatory Control *N* = 10, HU *N* = 8, RL *N* = 10; Old: Ambulatory Control *N* = 20, HU *N* = 10, RL *N* = 10). Soleus muscles were carefully teased apart using forceps then homogenized using a gentleMACS tissue dissociator (Miltenyi Biotec) at 37°C for 1 h. CD45+ cells were isolated as previously described using FACS (Reidy, McKenzie, et al., 2019). Briefly, the suspension obtained from homogenization was filtered through a 40 µm and then a second time through a 70 µm cell strainer. The remaining cell suspension was brought up in volume to 9 ml with F12 media. The suspension was spun and resuspended in 200 µl of FACS buffer (1% BSA‐PBS, pH 7.41). Prior to staining, a Fc receptor blocker (cat. no. 14‐0161‐82, eBioscience) was used for 5 min and then, afterward, incubated with anti‐CD45‐Percp. Cy7 (cat. no. 103114, Biolegend) antibody for 15 min in the dark. DAPI was added for 5 min for cell viability during cell sorting. The cell suspension was delivered on ice to the University of Utah Flow Cytometry Core and sorted for CD45+ DAPI−. The cells collected from the sort were pelleted and resuspended using the recommended 10× genomics buffer (0.1% BSA‐PBS) and then delivered on ice to the University of Utah Genomics Core. Paired‐end RNAseq (125 cycles) was performed via an 10× Chromium (10× Genomics) next‐generation sequencer. Approximately 1700–3700 cells per sample were sequenced with a viability of ~90%, and mean reads per cell ranged from 78,000 to 136,000.

### 2.4 Identification of cell clusters

Sequencing reads were processed by using 10× Genomics Cell Ranger pipeline and further analyzed with the Seurat and CIPR cluster analysis package in R (Ekiz et al., 2019, 2020). High and low mitochondrial read count cells were filtered (Segawa et al., 2008) from the data set prior to analysis (dead and or dying cells typically have abnormal mitochondrial read counts; Ekiz et al., 2019). Single cell sequencing clusters were identified as previously described (Ekiz et al., 2019, 2020). Briefly, cells were clustered by using FindClusters in the Seurat package. This approach identified clusters using the shared nearest neighbor (SNN) modularity algorithm. Identities of cell clusters were determined with the assistance of an immune cell scoring algorithm CIPR (Cluster Identity PRedictor) written in‐house by the second author (Ekiz et al., 2020). By surveying known immune cell markers in the SCseq data set and using differentially expressed gene signatures from Seurat, the immune‐scoring algorithm performed the following steps: (a) for each ImmGen cell population and for each gene found in ImmGen microarrays, it calculated the ratio of normalized microarray signal to the average signal value of the gene from the whole ImmGen data; (b) applied natural log transformation to the ratio, resulting in positive numbers for upregulated genes and negative numbers for downregulated genes in ImmGen data sets; (c) multiplied ImmGen log‐ratio values with the log‐ratio of matching genes that are differentially expressed in each cell cluster in the SCseq data set; and (d) summed scores from all the genes to yield an aggregate identity score for each ImmGen cell type for a given SCseq cluster. With this approach, genes that are differentially upregulated or downregulated in both ImmGen and SCseq data sets contribute to the immune identity score more heavily (a positive number is obtained when 2 log‐ratio values with the same sign are multiplied). In contrast, if a gene is inversely regulated in ImmGen and SCseq clusters, the immune identity score is reduced. Through this method, the correlation between the gene expression signatures of SCseq cell clusters in this study and ImmGen data subsets assisted in determining the cluster identities. In cases where this algorithm was unable to make a clear distinction (as in myeloid cell subsets), we surveyed the expression of known genes in the data set and performed differential expression analyses between closely related cell clusters. Upon naming the clusters, the Seurat R package was used to create plots for the expression of selected genes. GSEA analysis was performed by using fgsea R package, after ranking genes using a signal‐to‐noise metric. Raw sequence data can be obtained from the National Center for Biotechnology Information Gene Expression Omnibus repository entry GSE158987.

### 2.5 Bone marrow‐derived progenitor cell isolation and culturing of macrophages

The majority of macrophages found in muscle during stimuli such as reloading or damage/regeneration are a consequence of immune cell infiltration and commonly originate from the bone marrow (Grounds, 1987; Kanazawa et al., 2017). Therefore, as a follow‐up experiment, we isolated and cultured bone marrow progenitor cells from young (*N* = 4) and old (*N* = 4) mice at baseline (ambulatory control) and at 4 days of reloading following 14‐day hindlimb unloading. Following 7 days in M‐CSF, isolated cells were polarized to pro‐inflammatory macrophages, which we refer to as bone marrow‐derived macrophages; BMDM). To obtain BMDM, after completion of the mouse experimental treatments (control and reloading), mice were euthanized, and the femur and tibia bones dissected and cleaned of muscle and connective tissue. Bones were flushed using a 25‐gauge needle with sterile PBS into conical tubes and pelleted at 18 *g* for 5 min and then resuspended in red blood cell lysis buffer for 5 min (O'Connell et al., 2008). The remaining suspension containing bone marrow‐derived progenitor cells were spun down at 18 *g* for 5 min, resuspended using 8 ml of D10 medium (DMEM supplemented with 1% L‐glutamine, 10% FBS, 1% penicillin/streptomycin) and 10 ng/ml of mouse macrophage colony stimulating factor (M‐CSF,R&D Systems; O'Connell et al., 2008). After, cells were counted and plated in either 10‐cm plates at ~6–10 million cells/plate or slide (metabolomics) well chambers (phagocytosis) ~100,000 cells/plate for a period of 7 days to induce M0 macrophages status. Finally, macrophages (M0) were polarized to a pro‐inflammatory phenotype using a standard protocol of 100 ng/ml LPS and 50 ng/ml interferon gamma (IFNγ) for a duration of 24 h (Mounier et al., 2013).

### 2.6 Glycolytic and phagocytosis function

Following the procedure above, cells were counted and seeded in a Seahorse XF96 well plate at a density of 60,000 cells per well (Freemerman et al., 2014). Four replicate wells were seeded per treatment group for young and old mice. Polarized macrophages were subjected to a glycolysis stress test using the XF96 bioanalyzer at the University of Utah metabolic phenotyping core. Three key parameters of glycolytic function, glycolysis, glycolytic capacity, and glycolytic reserve were assessed during the test. Briefly, the real‐time bioenergetic profile was obtained by measuring the extracellular acidification rate using a Seahorse XF extracellular flux analyzer by adding glucose (10 mM final concentration), oligomycin (1 µM final concentration), and 2‐deoxy‐D‐glucose (100 mM; Van den Bossche et al., 2016). These three compounds were injected consecutively within a specific time gap, and extracellular acidification rate (ECAR) values were measured following each injection. Glycolysis, glycolytic capacity, and glycolytic reserve were calculated as previously described (Mookerjee et al., 2016). Cells were normalized to total protein content per well via Bradford method after completion of the Seahorse assay.

Phagocytosis capacity was performed using commercially available IgG FITC tagged latex bead kit (Cayman Chemical). Briefly, cells were seeded in slide well chambers at ~120,000 cells per well. FITC tagged beads were added at a dilution of 1:200 and incubated for 1h. Cells were washed using provided assay buffer and counterstained using WGA 649 (Alexaflour). Cells were imaged at 20× using a Nikon TI eclipse microscope, and beads were counted using ImageJ (NIH). Two hundred cells were analyzed per well (3 wells per treatment), and the average amount of beads per cell were displayed per treatment group as in indicator of phagocytosis function (Sikkema et al., 2018).

### 2.7 Targeted GC‐MS metabolomics

For metabolomics, bone marrow‐derived monocyte progenitor cells were isolated from young and old after 4 days of reloading (*N* = 4/age group) and then cultured and polarized to a pro‐inflammatory phenotype as described above. Cells were washed in ice cold PBS and pelleted. After, the cells were subjected to targeted GC‐MS metabolomics at the University of Utah Metabolomics Core. Intermediates of glycolysis and the TCA cycle were examined as detailed below.

Cold 90% methanol (MeOH) solution was added to each sample to give a final concentration of 80% MeOH to each cell pellet. Samples were incubated at −20°C for 1 h. After incubation, the samples were centrifuged at 20,000 *g* for 10 min at 4°C. The supernatant was then transferred from each sample tube into a labeled, fresh micro‐centrifuge tube. Pooled quality control samples were made by removing a fraction of the collected supernatant from each sample, while process blanks were made using only extraction solvent with no cell culture. The samples were dried in vacuo. All GC‐MS analyses were performed with an Agilent 5977b GC‐MS MSD‐HES and an Agilent 7693A automatic liquid sampler. Dried samples were suspended in 40 µl of a 40 mg/ml O‐methoxylamine hydrochloride (MOX; MP Bio #155405) in dry pyridine (EMD Millipore #PX2012‐7) and incubated for 1 h at 37℃ in a sand bath. Twenty‐five microliters of this solution was added to auto sampler vials. Sixty microliters of N‐methyl‐N‐trimethylsilyltrifluoracetamide (MSTFA with 1% TMCS, Thermo #TS48913) was added automatically via the auto sampler and incubated for 30 min at 37℃. After incubation, samples were vortexed and 1 µl of the prepared sample was injected into the gas chromatograph inlet in the split mode with the inlet temperature held at 250℃. A 5:1 split ratio was used for analysis for the majority of metabolites. Any metabolites that saturated the instrument at the 5:1 split were analyzed at a 50:1 split ratio. The gas chromatograph had an initial temperature of 60℃ for 1 min followed by a 10℃/min ramp to 325℃ and a hold time of 10 min. A 30‐meter Agilent Zorbax DB‐5MS with 10 m Duraguard capillary column was employed for chromatographic separation. Helium was used as the carrier gas at a rate of 1 ml/min. Data were collected using MassHunter software (Agilent). Metabolites were identified, and their peak area was recorded using MassHunter Quant. These data were transferred to an Excel spread sheet (Microsoft). Metabolite identity was established using a combination of an in‐house metabolite library developed using pure purchased standards, the NIST library and the Fiehn library.

### 2.8 Statistical analysis

Wilcoxon's test was used for comparing gene expression levels in scRNAseq data. GSEA analysis was performed using fgsea package in R, and *p*‐values were adjusted using Benjamini‐Hochberg method. Seahorse and phagocytosis data were analyzed using a *t* test (Prism Graphpad) at *p* < 0.05. For metabolomics, data were analyzed using in‐house software using the "MetaboAnalyst" software tool. Data were normalized by the median, transformed using generalized log transformation (glog 2), and scaled using Pareto scaling method. Clustering analysis was then performed via heatmap, and a univariate analysis was done using a volcano plot between young and old with the fold change cutoff being 1.5‐fold with a significant *p*‐value of 0.05. Metabolites above this threshold were considered significant on the univariate analysis and were listed in an accompanying table within Figure 5.

## 3 RESULTS

### 3.1 Single cell RNA sequencing of CD45+ cells and identification of pro‐inflammatory macrophages

**FIGURE 1:**
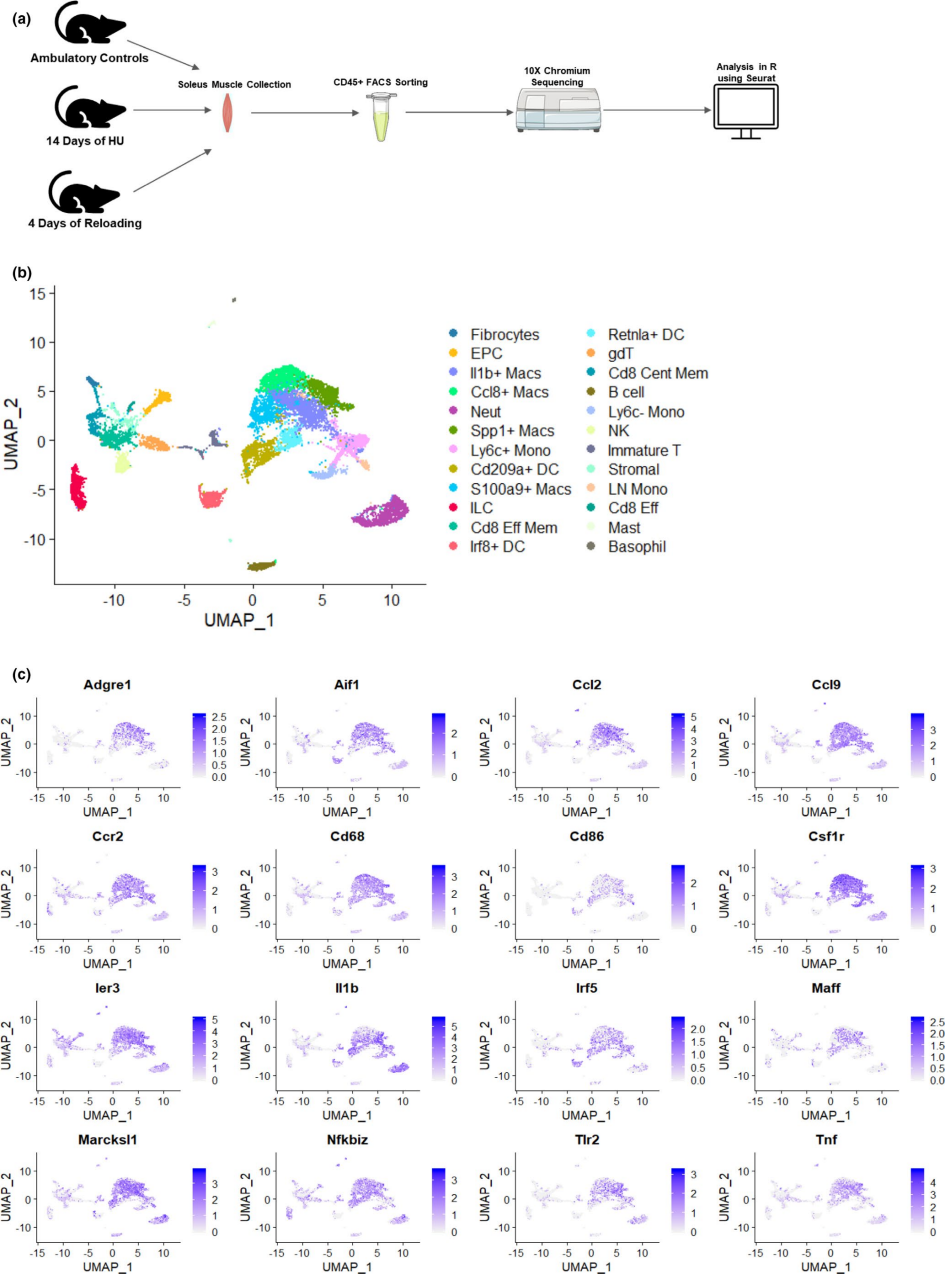
Experimental designs and single cell sequencing of CD45+ cells. (a) Study design depicting experimental groups, solei muscle collection, FACS, and single cell sequencing workflow. (b) UMAP supervised clustering of all CD45+ cell types identified by single cell RNA sequencing. Cell types identified include IL‐1β+, CCL8+, Spp1+, and S100a9+ macrophages, neutrophils, Ly6C+ and Ly6C‐ monocytes, Cd209+ dendritic cells, innate lymphoid cells, CD8 memory T cells, IRF8+ dendritic cells, Retnla dendritic cells, gamma delta T cells, endothelial progenitor cells (EPC), CD8 central memory T cells, B cells, natural killer cells, immature T cells, stromal cells, lymph node monocyte cells, fibrocytes, CD8 effector T cells, mast cells, and basophils. (c) UMAP expression of pan and pro‐inflammatory macrophage surface marker transcripts ADGRE1, AIF1, CCL2, CCL9, CCR2, CD68, CD86, CSF1R, IER3, Il1β, IRF5, MAFF, MARCKSL1, NFκBIZ, TLR2, and TNF. Panel A was generated using Servier Medical Art (SMART). Data depicted are from all treatment groups pooled together from both ages

Groups of young and old mice underwent 14 days of hindlimb unloading (HU) followed by 4 days of recovery (4‐day reloading). Soleus muscle weight between young and old was different at control, reduced with HU, and different at 4‐day reload between young and old with the soleus muscle from the old not fully recovered compared to baseline (Figure 1a). Soleus muscles were collected and pooled within each treatment group (Control, HU, 4‐day reload) for young and repeated for old mice equating to a total of six samples. Soleus samples were subjected to FACS and 10x Genomics scRNAseq. The workflow of the single cell sequencing experiment leading up to analysis in R using the Seurat package is depicted in Figure 1a. FACS was performed based on CD45 expression as this is a broadly expressed marker in all leukocyte populations. As a result, we identified a total of 24 unique cell clusters that were CD45+ ranging from T cells, B cells, neutrophils, dendritic cells, fibrocytes, monocytes, and natural killer cells (Figure 1b). We also note that the total CD45+ cells at each time point were similar between young and old (Supplemental Figure 1b,c). Using CIPR cluster analysis in R, we identified four macrophage populations across all the treatment groups. The only distinguishing feature within each macrophage population was higher expression of the cytokines and chemokines IL‐1β, Ccl8, Spp1, and S100a9. Upon further examination, we determined that these macrophage populations had no other unique feature from each other when evaluated across treatments and age (Figure 1b,c). We next utilized broad pan macrophage markers (CD68, CD86, Adgre1 (F4/80), CSF1r) to visualize total macrophages in the context of all total cells sequenced (Figure 1c). This was followed by utilizing markers indicative of a pro‐inflammatory macrophage phenotype (Aif1, Ccl2, Ccl9, Ccr2, Ier3, IL‐1β, Irf5, Maff, TLR2, Tnf, NFκB, Marcksl1; Orecchioni et al., 2019). These panel of markers demonstrated that these macrophages were predominately of the pro‐inflammatory origin. While we did capture some anti‐inflammatory markers in the macrophage populations including CD163, they were far less prevalent than pro‐inflammatory markers (Figure 1d) and are suggestive of the overlap and constantly dynamic state of macrophage populations. Given our interest in the recovery aspect of aged muscle and the lack of distinguishing features between these macrophage populations, we decided to combine the macrophage populations into a single cluster and focused on the recovery treatment group (day 4 reload) between the age groups for the remainder of analysis.

### 3.2 Inflammatory profile of aged macrophages is disrupted during 4 days of reloading following disuse

**FIGURE 2:**
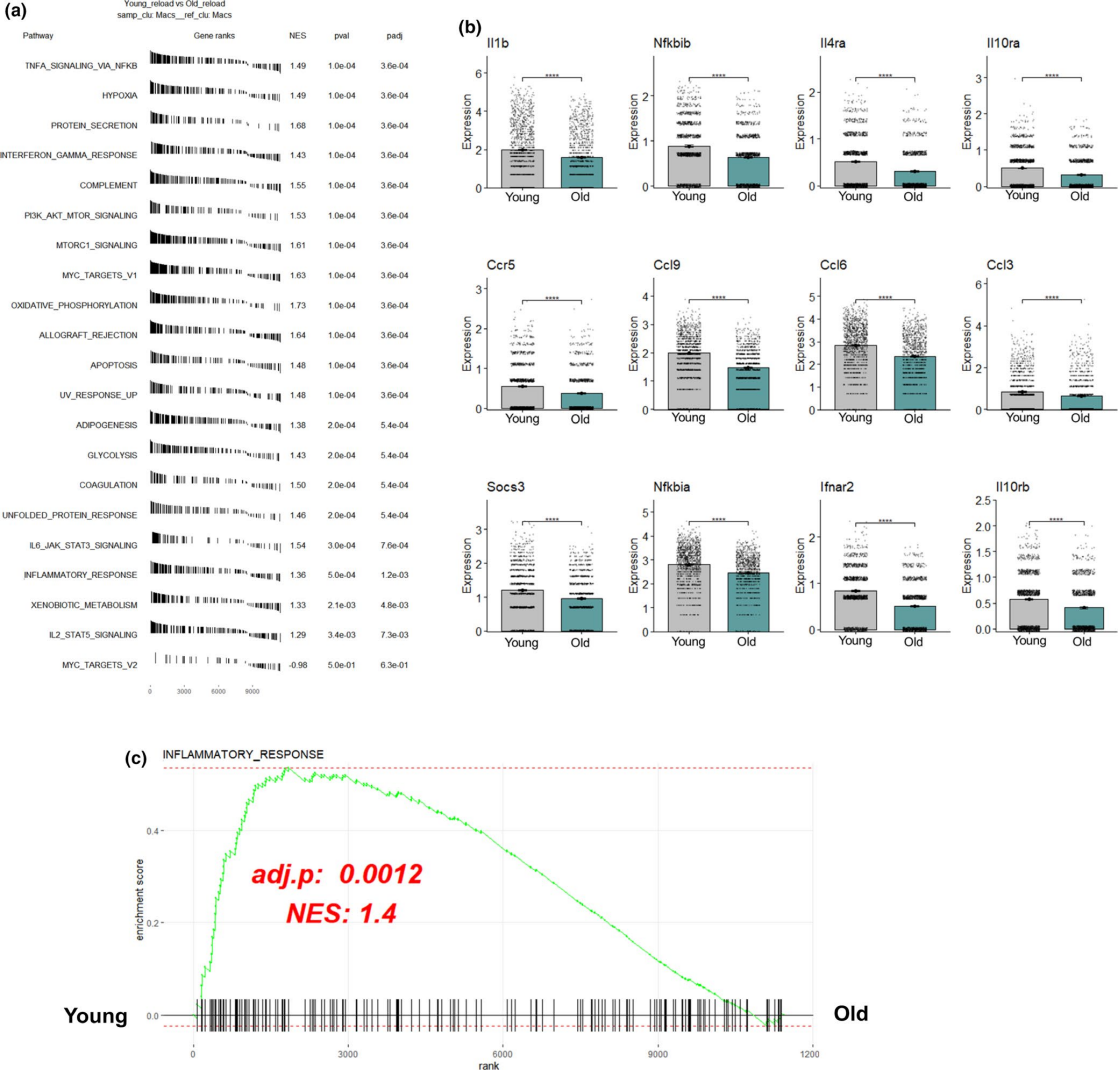
Inflammatory transcriptome of aged skeletal muscle macrophages is blunted after 4 days of reloading. (a) Gene set enrichment analysis (GSEA) of top regulated pathways from single cell sequencing transcripts in pro‐inflammatory macrophage clusters from day 4 of reloading after disuse. (b) Bar graphs of significantly different select inflammatory transcripts in young and old pro‐inflammatory macrophages from day 4 of reloading after disuse. (c) Individual GSEA analysis demonstrating enriched inflammatory signaling in young pro‐inflammatory macrophages from day 4 of reloading after disuse atrophy. NES denotes normalized enrichment score. A positive NES denotes enriched in young compared to old. Wilcoxon rank‐sum test was used to rank genes and determine significance for GSEA and boxplot analysis. Statistical significance is *p* < 0.05

The top pathways, using gene set enrichment analysis (GSEA), at the 4‐day reload treatment group demonstrated five inflammatory Hallmark pathways (TNFα Signaling via NFκB, Interferon Gamma Response, IL6 JAK STAT3 Signaling, Inflammatory Response, and IL2 STAT5 Signaling) that were enriched in the young macrophages when compared to old (Figure 2a). We next plotted represented inflammatory transcripts that are characteristic to pro‐inflammatory macrophages and in line with the GSEA pathways. Consistent with the GSEA analysis, aged pro‐inflammatory macrophages exhibited a blunted inflammatory transcriptome compared to young according to the listed inflammatory markers (Figure 2b). Of particular interest was the “Inflammatory Response” pathway that was enriched in the young reload when compared to old reload samples (Figure 2c). This pathway accounted for a bulk of cytokines and chemokines necessary to coordinate a proper inflammatory response within the tissue area of interest. We also show within the supplemental analysis that macrophages during the reload phase exhibit a higher “Inflammatory Response” when compared to the HU time point regardless of age which was expected due to the high volume of cells infiltrating during the reloading phase (Supplemental Figure 3). However, it is interesting to note that during the Control and HU conditions in the old (vs. young), GSEA inflammatory response was enriched (Supplemental Figures 2a and 3c) suggesting that old muscle macrophages exhibited a pro‐inflammatory phenotype at these time points which was impaired once reloading commenced.

### 3.3 Aged muscle macrophages exhibit an impaired glycolytic transcriptome during 4 days of reloading

**FIGURE 3:**
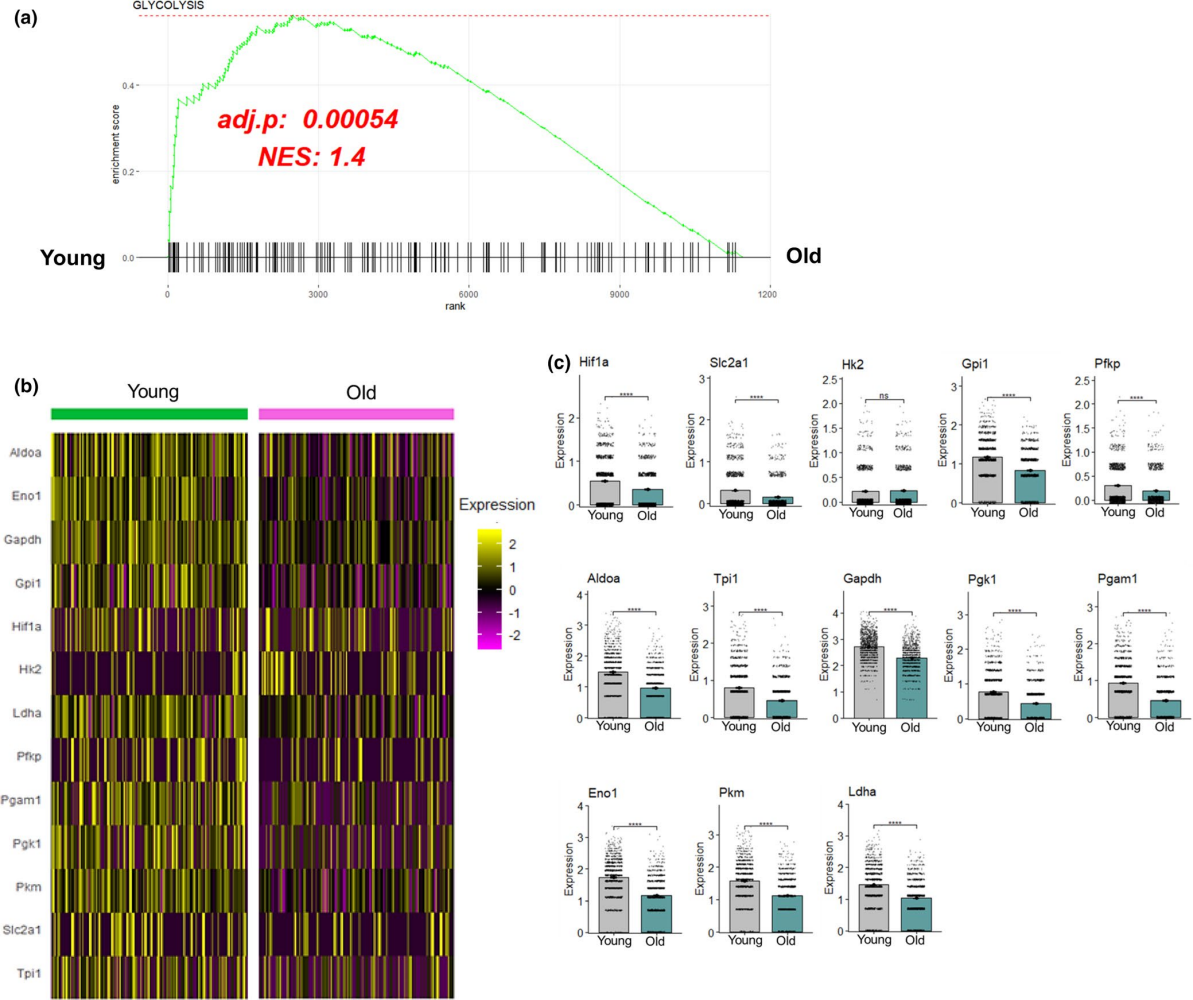
Aged skeletal muscle macrophages exhibit impaired glycolytic transcriptome during 4 days of reloading. (a) Individual GSEA analysis demonstrating enriched glycolysis pathways analysis in young pro‐inflammatory macrophages compared to old during 4 days of reloading from disuse. (b) Heatmap visualization of glycolytic enzyme transcripts Aldoa, Eno, Gapdh, Gpi‐1, Hif‐1α, Hk2, Ldha, Pfkp, Pgam1, Pgk, Pkm, Slc2a1 (Glut1), and Tpi‐1. (c) Bar graphs of glycolytic enzymes, Hif‐1α, and its immediate downstream target, Slc2a1 (Glut1). NES denotes normalized enrichment score. A positive NES denotes enriched in young compared to old. Wilcoxon rank‐sum test was used to rank genes and determine significance for GSEA and boxplot analysis. Statistical significance is *p* < 0.05

Muscle macrophages during reloading also included top pathways related to glycolysis and oxidative phosphorylation. Given that pro‐inflammatory macrophages are primarily glycolytic and inferred to regulate the inflammatory profile (Orecchioni et al., 2019), we elected to investigate the glycolytic phenotype further. Inspection of the individual GSEA analysis revealed an enriched glycolysis pathway in young macrophages during the reloading treatment (Figure 3a). Since HIF‐1α is understood to be a master regulator of glycolysis within pro‐inflammatory macrophages (Cramer et al., 2003), this was included within the analysis of glycolytic transcripts. We next utilized a heat map and individual bar graphs that showed a reduction in HIF‐1α in addition to its immediate known downstream target, Slc2a1 (Glut‐1; Freemerman et al., 2014), in aged macrophages compared to young (Figure 3b,c). Furthermore, every glycolytic enzyme transcript (except for Hexokinase 2 (HK2) was reduced compared to Young (Figure 3b,c) suggesting that the glycolytic program of aged pro‐inflammatory muscle macrophages was suppressed during recovery. Furthermore, the oxidative phosphorylation GSEA pathway and related transcripts were also lower in aged pro‐inflammatory macrophages inferring a potential disruption of mitochondria and the TCA cycle (Supplemental Figure 4a,b).

### 3.4 Glycolytic metabolism and phagocytosis in aged bone marrow‐derived macrophages following 4 days of reloading

**FIGURE 4:**
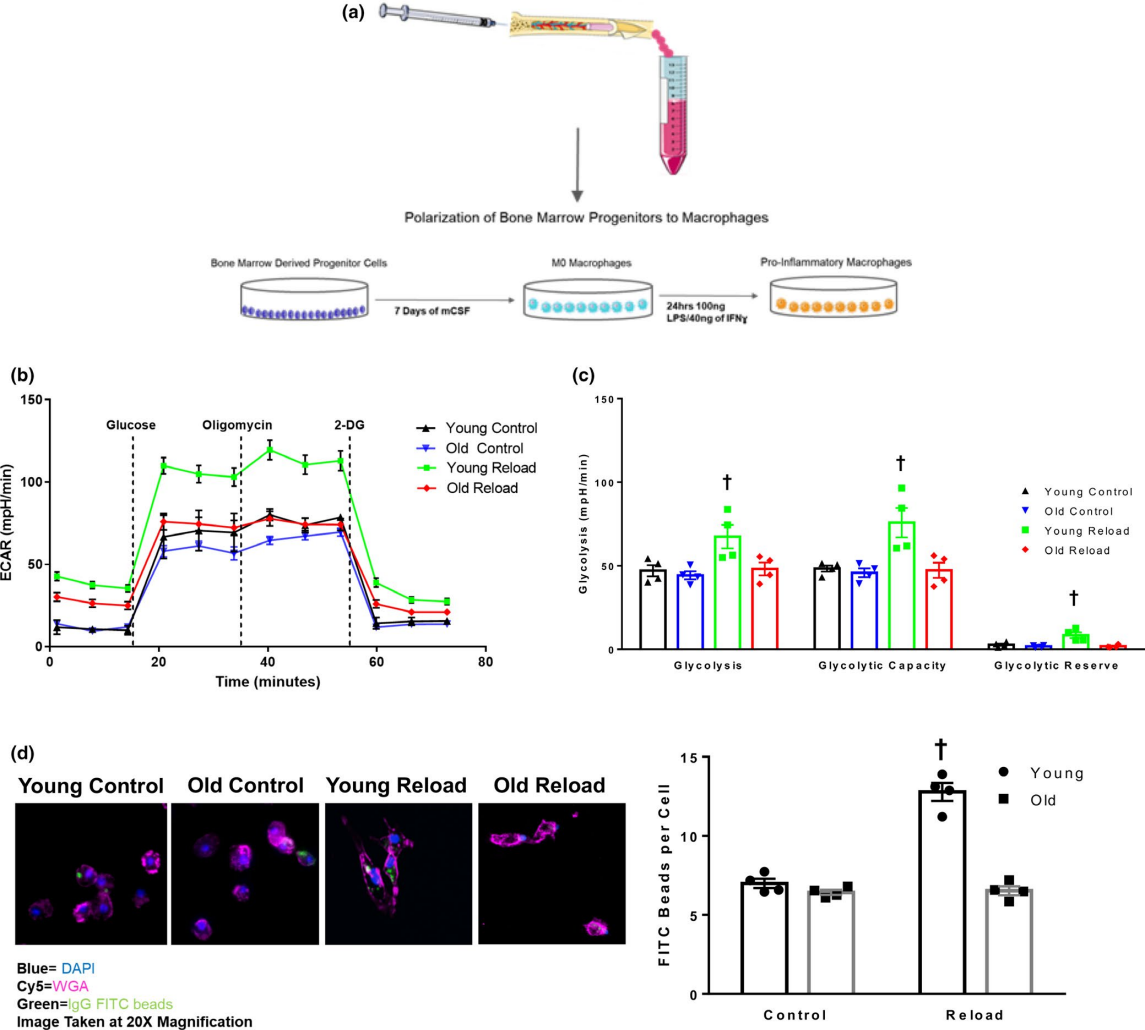
Impaired glycolytic metabolism and phagocytosis in aged bone marrow‐derived macrophages following 4 days of reloading. (a) Experimental design schematic showing workflow of bone marrow isolation and culturing of bone marrow progenitors and polarization to pro‐inflammatory macrophages. (b) Seahorse XFe96 bioanalyzer glycolysis stress test curves depicting ECAR at baseline, with glucose, oligomycin, and 2‐deoxyglucose in young and old control pro‐inflammatory macrophages and young and old 4‐day reload pro‐inflammatory macrophages. (c) Analysis of seahorse curves depicting glycolysis, glycolytic capacity, glycolytic reserve in young and old 4‐day reload pro‐inflammatory macrophages. (d) Images depicting young and old pro‐inflammatory macrophages after consuming IgG FITC tagged latex beads. Phagocytosis activity was graphed and expressed as average number of beads per cell (200 cells per group per replicate). Images were taken at 20× magnification. Panel a was generated using Servier Medical Art (SMART). Data are expressed as Mean ± SEM. Two‐way ANOVA used for analysis. *Represents different than Young (*p* < 0.05). ^†^Represents different from all groups (*p* < 0.05)

To further investigate the impaired glycolytic macrophage transcriptional signature between the age groups, we repeated the mouse disuse and 4‐day recovery experiments to examine the metabolic function in young and old macrophages during early recovery. Bone marrow‐derived progenitor cells at control and 4‐day RL were cultured and polarized to pro‐inflammatory macrophages (Figure 4a) and then subjected to a glycolysis stress test. As a result, old pro‐inflammatory macrophages (compared to young) had robustly lower glycolysis, glycolytic capacity, and glycolytic reserve (Figure 4b,c). We also demonstrated that suppressed glycolysis was distinctly present during the 4‐day reloading period in the old since glycolytic measurements on ambulatory control mouse macrophages were not different between young and old. This is also in agreement with the glycolytic‐related transcripts in young and old control muscle macrophages as well (data not shown).

Glycolysis has been demonstrated to be linked to pro‐inflammatory macrophage function and phagocytosis activity (Pavlou et al., 2018). Using IgG FITC tagged beads, we show increased phagocytosis in young bone marrow‐derived macrophages following 4 days of reloading but not in old bone marrow‐derived macrophages (Figure 4d). Overall, these results support that glycolytic metabolism and phagocytosis activity of aged pro‐inflammatory macrophages are impaired following 4 days of recovery following disuse atrophy.

### 3.5 Metabolome of aged bone marrow‐derived macrophages following 4 days of reloading

**FIGURE 5:**
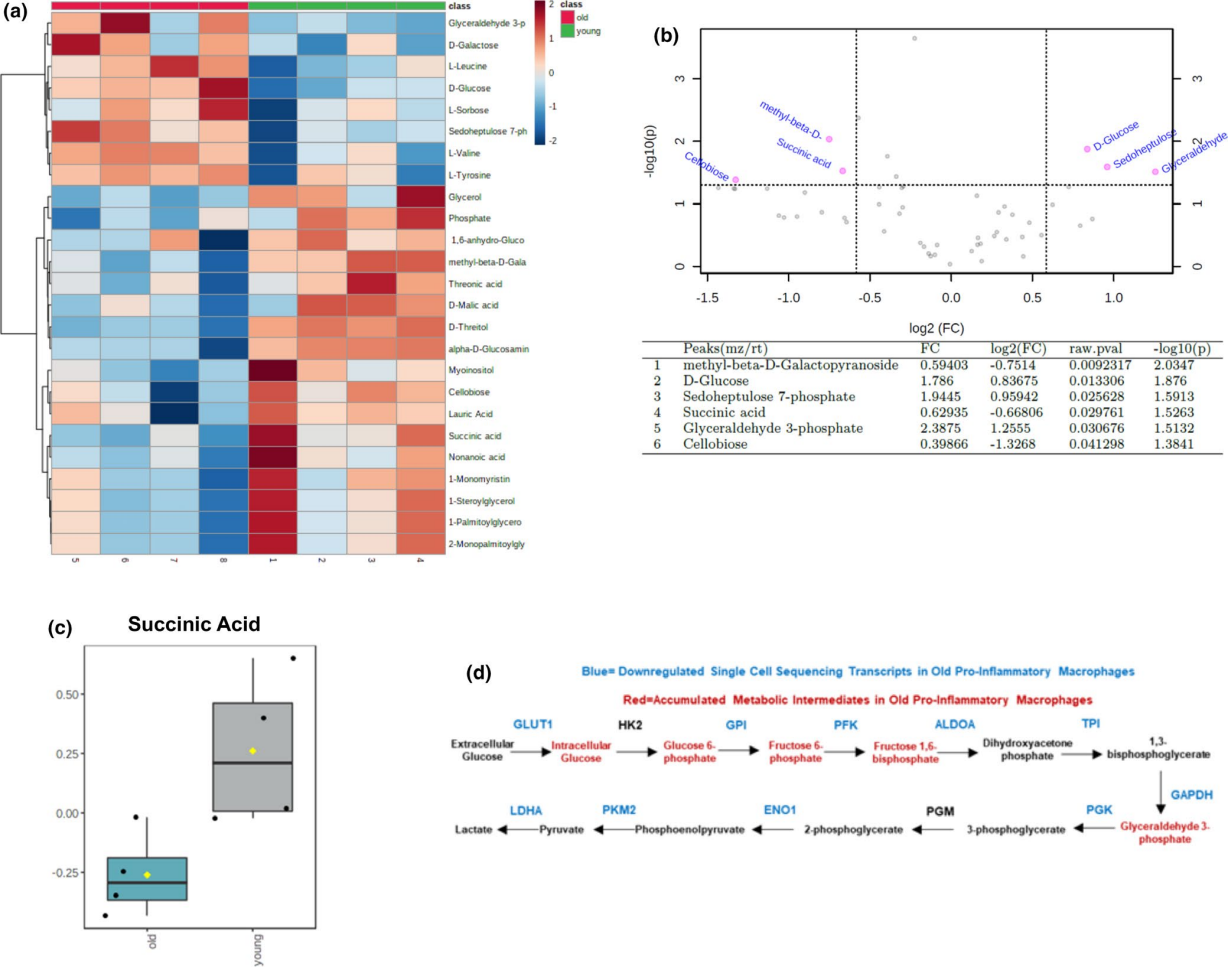
Metabolomic analysis of bone marrow‐derived macrophages following 4 days of reloading reveal a lack of succinate accumulation in aged mice. (a) Hierarchal clustering heatmap visualization of metabolites in young and old bone marrow‐derived pro‐inflammatory macrophages following 4 days of reloading. (b) Volcano plot and table of significant metabolites in young and old pro‐inflammatory bone marrow‐derived macrophages following 4 days of reloading. Dashed line represents fold change cutoff of 1.5 (anything above 1.5 with a *p*‐value of <0.05 was considered significant). (c) Boxplot of succinic acid in young and old pro‐inflammatory bone marrow‐derived macrophages following 4 days of reloading, yellow dot denotes the mean of each group. (d) Summary glycolysis panel highlighting outcomes observed from the metabolomic and single cell sequencing data. Blue text denotes downregulated transcripts as determined by single cell sequencing in old pro‐inflammatory macrophages obtained from soleus muscle. Red text denotes accumulated metabolite intermediates in old pro‐inflammatory macrophages isolated from bone marrow. Heatmap was created using MetaboAnalyst Hierarchal clustering. Volcano plot data and succinic acid boxplot was analyzed using univariate analysis in MetaboAnalyst software. Statistical significance was *p* < 0.05

Finally, to examine the metabolic deficit in old pro‐inflammatory macrophages, we subjected a new cohort of polarized pro‐inflammatory cells (collected in a similar manner as in experiment Figure 4a) to targeted GC‐MS metabolomic analysis for glycolysis and TCA cycle intermediate assessment. In old macrophages, we show, using hierarchal clustering analysis, an accumulation of glycolytic intermediates and a lack of an increase in the classical TCA intermediates associated with pro‐inflammatory macrophage metabolism (Figure 5a). Interestingly, old (compared to young) pro‐inflammatory macrophages were characterized with a decrease in the TCA intermediate, succinic acid, which has been shown to be a key driver of HIF‐1α‐mediated inflammatory and glycolytic reprogramming (Figure 5b,c). Furthermore, glucose, G3P, and sedoheptulose 7‐phosphate were accumulated in old pro‐inflammatory macrophages suggestive of an impaired glycolysis and enhanced pentose phosphate pathway response (Figure 5b). Together, the targeted metabolomic analysis, in combination with the glycolytic transcript data, supports an age‐related disruption of intermediates that are associated with the glycolysis pathway (Figure 5d). Additionally, in contrast to the classical metabolome seen in the younger cells during muscle recovery, aged pro‐inflammatory macrophages exhibit a blunted succinic acid response inferring an impaired TCA cycle.

## 4 DISCUSSION

Recent evidence has highlighted the importance of macrophages in the regrowth of skeletal muscle following disuse atrophy and that macrophage function is tightly coupled to their metabolic phenotype (O'Neill & Artyomov, 2019; Reidy, McKenzie, et al., 2019). We sought to thoroughly characterize isolated muscle macrophages in aged muscle during early recovery from disuse using single cell RNA sequencing and metabolic functional approaches to gain insight in the relationship between macrophage polarization and the metabolic profile. Using single cell transcriptomics, we found that macrophage inflammatory and glycolysis transcripts were dysregulated during early recovery from disuse atrophy in aged muscle. Furthermore, we also report that aged macrophages isolated from bone marrow progenitor cells (compared to young) after 4 days of recovery are characterized with impaired glycolytic metabolism, phagocytosis, and lower succinate levels. Together, these results suggest that the metabolism of aged pro‐inflammatory macrophages is disrupted during the early recovery phase following disuse and this may contribute to the poor regrowth of aged skeletal muscle.

Using 10× Genomics single cell RNA sequencing on young and old leukocytes (CD45+), we observed a clear age discrepancy in the inflammatory profile of infiltrated pro‐inflammatory macrophages during early recovery following disuse atrophy such that aged macrophages had a consistently lower inflammatory status than young. This is contrary to the elevated inflammatory transcriptome present in resident aged pro‐inflammatory macrophages at baseline. When compared to their younger counterparts, these data suggest that the invading macrophages during the reloading phase may have a weak or potentially delayed inflammatory response. It is worth considering that the elevated intrinsic baseline inflammatory transcriptome of old macrophages may alter the sensitivity of these cells to the microenvironment thus impairing the ability to mount a proper immune response during the reloading phase of muscle regrowth. It is also possible that the dysregulated bone marrow‐derived macrophages invading reloaded muscle may be contributing to the dysfunction of the aged muscle microenvironment. Our findings during muscle reloading in aged mice are consistent with others that have observed a lower inflammatory response in macrophages following acute muscle injury in aging and in our previous work showing a reduced macrophage response in the old following disuse atrophy (Chazaud et al., 2009; Reidy, McKenzie, et al., 2019; Tidball & St Pierre, 1996). While we do recognize that reloading does not produce pronounced damage as injury models, aged muscle is quite susceptible to excessive damage following disuse atrophy (Kanazawa et al., 2017). Thus, the data generated above provide valuable insight into the dysregulated inflammatory response during the early reloading process in aged skeletal muscle.

The importance of the pro‐inflammatory response of macrophages during muscle recovery is evident. In the presence of damage, these specialized cells are critical for the secretion of pro‐inflammatory cytokines, chemokines, and enzymes such as cyclooxygenase‐2 (COX‐2) to stimulate myogenic precursor cell function and aide in muscle regrowth and regeneration (Dort et al., 2019; Ho et al., 2017; Palla et al., 2021; Shen et al., 2006). Within hours of a stimuli, neutrophils infiltrate the tissue to mount an immune response which serves to aid in pro‐inflammatory macrophage recruitment (Arnold et al., 2007). Pro‐inflammatory macrophages secrete cytokines such as CCL2, IL‐1β, TNF‐α, and IFN‐γ that, in culture, increase myoblast proliferation (Otis et al., 2014). Macrophage inflammation has been demonstrated to directly contribute to satellite cell activation and drive the skeletal muscle regeneration response (Cantini et al., 1994; Du et al., 2017). Myeloid depletion studies following injury document reduced phagocytosis capacity, proliferation of satellite cells, and the seeding of new myofibers (Segawa et al., 2008). However, in the current study, it is important to acknowledge that only one early time point was examined during recovery and the macrophage inflammatory response observed in these old mouse macrophages could also be delayed or have a lower polarization rate. Regardless, interruption in the timing or polarization of macrophage states severely compromises muscle recovery and myogenic cell function (Tidball & Wehling‐Henricks, 2007) clearly emphasizing that proper macrophage phenotype transition and function over the time course of regrowth from disuse is critical for optimal resolution of skeletal muscle. Together, these data suggest that pro‐inflammatory macrophages in aged skeletal muscle exhibit a dysregulated inflammatory transcriptome possibly related to impaired muscle regrowth following disuse atrophy.

Macrophage function and inflammatory status is reliant on metabolic reprogramming (Freemerman et al., 2014; Yoon et al., 2018). In the current study, we demonstrated that aged muscle pro‐inflammatory macrophages were characterized with lower expression of transcripts related to glycolysis and HIF‐1α during early recovery. Furthermore, we also determined that glycolysis and phagocytosis function were reduced in pro‐inflammatory macrophages derived from bone marrow progenitor cells of aged mice during recovery from disuse. Classically activated pro‐inflammatory macrophages rely mainly on glycolysis, pentose phosphate pathway, and lipid synthesis (Viola et al., 2019). Additionally, pro‐inflammatory macrophages are characterized with two breaks in the TCA cycle that result in the accumulation of itaconate and succinate (O'Neill, 2015). The pro‐inflammatory transcription factor, NF‐κB, and succinate prevent the degradation of HIF‐1α thereby allowing transcriptional upregulation of glycolytic and inflammatory genes (Cramer et al., 2003). Loss of HIF‐1α in myeloid cells impairs macrophage inflammation and skeletal muscle regeneration in a rodent model of acute muscle injury (Yang et al., 2017). Furthermore, in another study, COX‐2 was reduced in myeloid deficient HIF‐1α muscle suggesting that HIF‐1α is essential for macrophage‐mediated inflammation (Cramer et al., 2003). Finally, HIF‐1α promotes the conversion of pyruvate to lactate by controlling the expression of lactate dehydrogenase and pyruvate dehydrogenase kinase thereby limiting pyruvate from entering the Krebs cycle (Juban & Chazaud, 2017; Thapa & Lee, 2019). Together, these results support that the impairment of glycolysis in aged pro‐inflammatory muscle macrophages may be mediated in part by reduced HIF‐1α transcriptional activity, but this remains to be determined.

Another important finding was that succinate was elevated in young pro‐inflammatory macrophages but not aged pro‐inflammatory macrophages during recovery from disuse. The reprogramming of pro‐inflammatory macrophages to adopt a glycolytic function is dependent on the accumulation of succinate (Diskin & Palsson‐McDermott, 2018). Succinate has been recognized as not only a metabolic intermediate but also an important signaling molecule that regulates inflammatory status in macrophages (Mills et al., 2016). For example, LPS stimulation of pro‐inflammatory macrophages induced succinate accumulation, HIF‐1α protein expression, and increased inflammation mediated through IL‐1β (Tannahill et al., 2013). Furthermore, in the current data set, aged pro‐inflammatory macrophages isolated from muscle also were characterized with a disrupted oxidative phosphorylation transcriptional profile suggesting that an impaired succinate response in old macrophages during recovery may be due to mitochondrial dysfunction upon activation of the pro‐inflammatory state. This is consistent with a recent muscle recovery study in which whole muscle from aged mice was described by mitochondrial dysfunction and reduced TCA intermediates (Zhang et al., 2018). While we recognize that caution should be used when comparing macrophages derived from bone marrow progenitors (metabolic experiments) and muscle tissue macrophages (single cell transcriptomics), it is well recognized that the majority of macrophages during regeneration or regrowth are a consequence of invasion that most likely originate from bone marrow (Sun et al., 2009). We also recognize that the metabolomic data produced a diverse metabolic phenotype especially in the young which is most likely a consequence of the dynamic macrophage response and the difficulty of capturing the full metabolomic phenotype in culture. Further experimentation will be required to provide mechanistic insight as to why aged macrophages fail to accumulate succinate and a robust metabolomic profile during early muscle recovery.

In summary, this unique data set using 10× single cell RNA sequencing and metabolic and functional analysis demonstrate that aging disrupts the classical skeletal muscle pro‐inflammatory macrophage glycolytic phenotype and function during early recovery following disuse. Interestingly, aged pro‐inflammatory macrophages from skeletal muscle exhibited a lower inflammatory and glycolytic transcriptome that coincided with an impaired HIF‐1α transcriptional response. Glycolytic function and phagocytosis of aged pro‐inflammatory bone marrow‐derived macrophages during early recovery were also reduced. Metabolomic analysis confirmed an age‐specific impairment of flux through the glycolytic pathway and a dysfunctional TCA cycle marked by a lack of succinate accumulation. Overall, the evidence of reduced succinate, lower HIF‐1α transcription, and suppressed glycolysis suggest that aging dysregulates the metabolic reprogramming of skeletal muscle pro‐inflammatory macrophages during the early recovery phase following disuse (Graphical Abstract).

## CONFLICT OF INTEREST

None declared.

## AUTHOR CONTRIBUTIONS

M.J.D and R.M.O designed the research; D.K.F performed the research; D.K.F and A.E. conducted data analyses. D.K.F., J.J.P., A.I.M., and Z.S.M. contributed to data collection; D.K.F. and M.J.D. wrote the manuscript. All authors contributed to editing the manuscript.

## Supporting information

Fig S1

Fig S2

Fig S3

Fig S4

## Data Availability

The data from this study are available from the corresponding author upon reasonable request.

## References

[acel13448-bib-0001] Arnold, L., Henry, A., Poron, F., Baba‐Amer, Y., van Rooijen, N., Plonquet, A., Gherardi, R. K., & Chazaud, B. (2007). Inflammatory monocytes recruited after skeletal muscle injury switch into antiinflammatory macrophages to support myogenesis. Journal of Experimental Medicine, 204(5), 1057–1069. 10.1084/jem.20070075 PMC2118577 17485518

[acel13448-bib-0002] Cantini, M., Massimino, M. L., Bruson, A., Catani, C., Dallalibera, L., & Carraro, U. (1994). Macrophages regulate proliferation and differentiation of satellite cells. Biochemical and Biophysical Research Communications, 202(3), 1688–1696. 10.1006/bbrc.1994.2129 8060358

[acel13448-bib-0003] Chazaud, B. (2020). Inflammation and skeletal muscle regeneration: Leave it to the macrophages! Trends in Immunology, 41(6), 481–492. 10.1016/j.it.2020.04.006 32362490

[acel13448-bib-0004] Chazaud, B., Brigitte, M., Yacoub‐Youssef, H., Arnold, L., Gherardi, R., Sonnet, C., Lafuste, P., & Chretien, F. (2009). Dual and beneficial roles of macrophages during skeletal muscle regeneration. Exercise and Sport Sciences Reviews, 37(1), 18–22. 10.1097/JES.0b013e318190ebdb 19098520

[acel13448-bib-0005] Cramer, T., Yamanishi, Y., Clausen, B. E., Forster, I., Pawlinski, R., Mackman, N., & Johnson, R. S. (2003). HIF‐1alpha is essential for myeloid cell‐mediated inflammation. Cell, 112(5), 645–657. 10.1016/s0092-8674(03)00154-5 12628185 PMC4480774

[acel13448-bib-0006] Cui, C. Y., Driscoll, R. K., Piao, Y. L., Chia, C. W., Gorospe, M., & Ferrucci, L. (2019). Skewed macrophage polarization in aging skeletal muscle. Aging Cell, 18(6), e13032. 10.1111/acel.13032 31478346 PMC6826159

[acel13448-bib-0007] Diskin, C., & Palsson‐McDermott, E. M. (2018). Metabolic modulation in macrophage effector function. Frontiers in Immunology, 9, 270. 10.3389/fimmu.2018.00270 29520272 PMC5827535

[acel13448-bib-0008] Dort, J., Fabre, P., Molina, T., & Dumont, N. A. (2019). Macrophages are key regulators of stem cells during skeletal muscle regeneration and diseases. Stem Cells International, 2019, 4761427. 10.1155/2019/4761427 31396285 PMC6664695

[acel13448-bib-0009] Du, H., Shih, C.‐H., Wosczyna, M. N., Mueller, A. A., Cho, J., Aggarwal, A., Rando, T. A., & Feldman, B. J. (2017). Macrophage‐released ADAMTS1 promotes muscle stem cell activation. Nature Communications, 8, 669. 10.1038/s41467-017-00522-7 PMC5610267 28939843

[acel13448-bib-0010] Duchesne, E., Dufresne, S. S., & Dumont, N. A. (2017). Impact of inflammation and anti‐inflammatory modalities on skeletal muscle healing: From fundamental research to the clinic. Physical Therapy, 97(8), 807–817. 10.1093/ptj/pzx056 28789470

[acel13448-bib-0011] Ekiz, H. A., Conley, C. J., Stephens, W. Z., & O'Connell, R. M. (2020). CIPR: A web‐based R/shiny app and R package to annotate cell clusters in single cell RNA sequencing experiments. BMC Bioinformatics, 21(1), 191. 10.1186/s12859-020-3538-2 32414321 PMC7227235

[acel13448-bib-0012] Ekiz, H. A., Huffaker, T. B., Grossmann, A. H., Stephens, W. Z., Williams, M. A., Round, J. L., & O'Connell, R. M. (2019). MicroRNA‐155 coordinates the immunological landscape within murine melanoma and correlates with immunity in human cancers. JCI Insight, 4(6), e126543. 10.1172/jci.insight.126543 PMC6482995 30721153

[acel13448-bib-0013] Freemerman, A. J., Johnson, A. R., Sacks, G. N., Milner, J. J., Kirk, E. L., Troester, M. A., Macintyre, A. N., Goraksha‐Hicks, P., Rathmell, J. C., & Makowski, L. (2014). Metabolic reprogramming of macrophages: Glucose transporter 1 (GLUT1)‐mediated glucose metabolism drives a proinflammatory phenotype. Journal of Biological Chemistry, 289(11), 7884–7896. 10.1074/jbc.M113.522037 PMC3953299 24492615

[acel13448-bib-0014] Grounds, M. D. (1987). Phagocytosis of necrotic muscle in muscle isografts is influenced by the strain, age, and sex of host mice. Journal of Pathology, 153(1), 71–82. 10.1002/path.1711530110 3668737

[acel13448-bib-0015] Ho, A. T. V., Palla, A. R., Blake, M. R., Yucel, N. D., Wang, Y. X., Magnusson, K. E. G., Holbrook, C. A., Kraft, P. E., Delp, S. L., & Blau, H. M. (2017). Prostaglandin E2 is essential for efficacious skeletal muscle stem‐cell function, augmenting regeneration and strength. Proceedings of the National Academy of Sciences of the United States of America, 114(26), 6675–6684. 10.1073/pnas.1705420114 28607093 PMC5495271

[acel13448-bib-0016] Jing, C., Castro‐Dopico, T., Richoz, N., Tuong, Z. K., Ferdinand, J. R., Lok, L. S. C., Loudon, K. W., Banham, G. D., Mathews, R. J., Cader, Z., Fitzpatrick, S., Bashant, K. R., Kaplan, M. J., Kaser, A., Johnson, R. S., Murphy, M. P., Siegel, R. M., & Clatworthy, M. R. (2020). Macrophage metabolic reprogramming presents a therapeutic target in lupus nephritis. Proceedings of the National Academy of Sciences of the United States of America, 117(26), 15160–15171. 10.1073/pnas.2000943117 32541026 PMC7334513

[acel13448-bib-0017] Juban, G., & Chazaud, B. (2017). Metabolic regulation of macrophages during tissue repair: Insights from skeletal muscle regeneration. FEBS Letters, 591(19), 3007–3021. 10.1002/1873-3468.12703 28555751

[acel13448-bib-0018] Kanazawa, Y., Ikegami, K., Sujino, M., Koinuma, S., Nagano, M., Oi, Y., Onishi, T., Sugiyo, S., Takeda, I., Kaji, H., & Shigeyoshi, Y. (2017). Effects of aging on basement membrane of the soleus muscle during recovery following disuse atrophy in rats. Experimental Gerontology, 98, 153–161. 10.1016/j.exger.2017.08.014 28803135

[acel13448-bib-0019] Mills, E. L., Kelly, B., Logan, A., Costa, A. S. H., Varma, M., Bryant, C. E., Tourlomousis, P., Däbritz, J. H. M., Gottlieb, E., Latorre, I., Corr, S. C., McManus, G., Ryan, D., Jacobs, H. T., Szibor, M., Xavier, R. J., Braun, T., Frezza, C., Murphy, M. P., & O'Neill, L. A. (2016). Succinate dehydrogenase supports metabolic repurposing of mitochondria to drive inflammatory macrophages. Cell, 167(2), 457–470.e413. 10.1016/j.cell.2016.08.064 27667687 PMC5863951

[acel13448-bib-0020] Mookerjee, S. A., Nicholls, D. G., & Brand, M. D. (2016). Determining maximum glycolytic capacity using extracellular flux measurements. PLoS One, 11(3), e0152016. 10.1371/journal.pone.0152016 27031845 PMC4816457

[acel13448-bib-0021] Mounier, R., Theret, M., Arnold, L., Cuvellier, S., Bultot, L., Goransson, O., & Chazaud, B. (2013). AMPKalpha1 regulates macrophage skewing at the time of resolution of inflammation during skeletal muscle regeneration. Cell Metabolism, 18(2), 251–264. 10.1016/j.cmet.2013.06.017 23931756

[acel13448-bib-0022] O'Connell, R. M., Rao, D. S., Chaudhuri, A. A., Boldin, M. P., Taganov, K. D., Nicoll, J., Paquette, R. L., & Baltimore, D. (2008). Sustained expression of microRNA‐155 in hematopoietic stem cells causes a myeloproliferative disorder. Journal of Experimental Medicine, 205(3), 585–594. 10.1084/jem.20072108 PMC2275382 18299402

[acel13448-bib-0023] O'Neill, L. A. (2015). A broken krebs cycle in macrophages. Immunity, 42(3), 393–394. 10.1016/j.immuni.2015.02.017 25786167

[acel13448-bib-0024] O'Neill, L. A. J., & Artyomov, M. N. (2019). Itaconate: The poster child of metabolic reprogramming in macrophage function. Nature Reviews Immunology, 19(5), 273–281. 10.1038/s41577-019-0128-5 30705422

[acel13448-bib-0025] Orecchioni, M., Ghosheh, Y., Pramod, A. B., & Ley, K. (2019). Macrophage polarization: Different gene signatures in M1(LPS+) vs. classically and M2(LPS−) vs. alternatively activated macrophages. Frontiers in Immunology, 10, 1084. 10.3389/fimmu.2019.01084 31178859 PMC6543837

[acel13448-bib-0026] Otis, J. S., Niccoli, S., Hawdon, N., Sarvas, J. L., Frye, M. A., Chicco, A. J., & Lees, S. J. (2014). Pro‐inflammatory mediation of myoblast proliferation. PLoS One, 9(3), e92363. 10.1371/journal.pone.0092363 24647690 PMC3960233

[acel13448-bib-0027] Palla, A. R., Ravichandran, M., Wang, Y. X., Alexandrova, L., Yang, A. V., Kraft, P., Holbrook, C. A., Schürch, C. M., Ho, A. T. V., & Blau, H. M. (2021). Inhibition of prostaglandin‐degrading enzyme 15‐PGDH rejuvenates aged muscle mass and strength. Science, 371(6528), eabc8059. 10.1126/science.abc8059 33303683 PMC7938328

[acel13448-bib-0028] Pavlou, S., Lindsay, J., Ingram, R., Xu, H., & Chen, M. (2018). Sustained high glucose exposure sensitizes macrophage responses to cytokine stimuli but reduces their phagocytic activity. BMC Immunology, 19(1), 24. 10.1186/s12865-018-0261-0 29996768 PMC6042333

[acel13448-bib-0029] Reidy, P. T., Dupont‐Versteegden, E. E., & Drummond, M. J. (2019). Macrophage regulation of muscle regrowth from disuse in aging. Exercise and Sport Sciences Reviews, 47(4), 246–250. 10.1249/JES.0000000000000201 31525165 PMC9236178

[acel13448-bib-0030] Reidy, P. T., McKenzie, A. I., Mahmassani, Z. S., Petrocelli, J. J., Nelson, D. B., Lindsay, C. C., Gardner, J. E., Morrow, V. R., Keefe, A. C., Huffaker, T. B., Stoddard, G. J., Kardon, G., O'Connell, R. M., & Drummond, M. J. (2019). Aging impairs mouse skeletal muscle macrophage polarization and muscle‐specific abundance during recovery from disuse. American Journal of Physiology‐Endocrinology and Metabolism, 317(1), E85–E98. 10.1152/ajpendo.00422.2018 30964703 PMC6689737

[acel13448-bib-0031] Segawa, M., Fukada, S., Yamamoto, Y., Yahagi, H., Kanematsu, M., Sato, M., Ito, T., Uezumi, A., Hayashi, S., & Miyagoesuzuki, Y. (2008). Suppression of macrophage functions impairs skeletal muscle regeneration with severe fibrosis. Experimental Cell Research, 314(17), 3232–3244. 10.1016/j.yexcr.2008.08.008 18775697

[acel13448-bib-0032] Shen, W., Prisk, V., Li, Y., Foster, W., & Huard, J. (2006). Inhibited skeletal muscle healing in cyclooxygenase‐2 gene‐deficient mice: The role of PGE2 and PGF2alpha. Journal of Applied Physiology, 101(4), 1215–1221. 10.1152/japplphysiol.01331.2005 16778000

[acel13448-bib-0033] Sikkema, A. H., Stoffels, J. M. J., Wang, P., Basedow, F. J., Bulsink, R., Bajramovic, J. J., & Baron, W. (2018). Fibronectin aggregates promote features of a classically and alternatively activated phenotype in macrophages. Journal of Neuroinflammation, 15(1), 218. 10.1186/s12974-018-1238-x 30071854 PMC6091019

[acel13448-bib-0034] Sun, D., Martinez, C. O., Ochoa, O., Ruiz‐Willhite, L., Bonilla, J. R., Centonze, V. E., Waite, L. L., Michalek, J. E., McManus, L. M., & Shireman, P. K. (2009). Bone marrow‐derived cell regulation of skeletal muscle regeneration. The FASEB Journal, 23(2), 382–395. 10.1096/fj.07-095901 18827026 PMC2630778

[acel13448-bib-0035] Tannahill, G. M., Curtis, A. M., Adamik, J., Palsson‐McDermott, E. M., McGettrick, A. F., Goel, G., & O'Neill, L. A. (2013). Succinate is an inflammatory signal that induces IL‐1beta through HIF‐1alpha. Nature, 496(7444), 238–242. 10.1038/nature11986 23535595 PMC4031686

[acel13448-bib-0036] Thapa, B., & Lee, K. (2019). Metabolic influence on macrophage polarization and pathogenesis. BMB Reports, 52(6), 360–372. 10.5483/BMBRep.2019.52.6.140 31186085 PMC6605523

[acel13448-bib-0037] Tidball, J. G., & St Pierre, B. A. (1996). Apoptosis of macrophages during the resulution of muscle inflammation. Journal of Leukocyte Biology, 59(3), 380–388. 10.1002/jlb.59.3.380 8604016

[acel13448-bib-0038] Tidball, J. G., & Wehling‐Henricks, M. (2007). Macrophages promote muscle membrane repair and muscle fibre growth and regeneration during modified muscle loading in mice in vivo. Journal of Physiology, 578(Pt 1), 327–336. 10.1113/jphysiol.2006.118265 PMC2075127 17038433

[acel13448-bib-0039] Van den Bossche, J., Baardman, J., Otto, N. A., van der Velden, S., Neele, A. E., van den Berg, S. M., Luque‐Martin, R., Chen, H.‐J., Boshuizen, M. C. S., Ahmed, M., Hoeksema, M. A., de Vos, A. F. , & de Winther, M. P. J. (2016). Mitochondrial dysfunction prevents repolarization of inflammatory macrophages. Cell Reports, 17(3), 684–696. 10.1016/j.celrep.2016.09.008 27732846

[acel13448-bib-0040] Viola, A., Munari, F., Sanchez‐Rodriguez, R., Scolaro, T., & Castegna, A. (2019). The metabolic signature of macrophage responses. Frontiers in Immunology, 10, 1462. 10.3389/fimmu.2019.01462 31333642 PMC6618143

[acel13448-bib-0041] Wang, Y., Wehling‐Henricks, M., Samengo, G., & Tidball, J. G. (2015). Increases of M2a macrophages and fibrosis in aging muscle are influenced by bone marrow aging and negatively regulated by muscle‐derived nitric oxide. Aging Cell, 14(4), 678–688. 10.1111/acel.12350 26009878 PMC4531081

[acel13448-bib-0042] Wang, Y., Wehling‐Henricks, M., Welc, S. S., Fisher, A. L., Zuo, Q., & Tidball, J. G. (2019). Aging of the immune system causes reductions in muscle stem cell populations, promotes their shift to a fibrogenic phenotype, and modulates sarcopenia. The FASEB Journal, 33(1), 1415–1427. 10.1096/fj.201800973R 30130434 PMC6355087

[acel13448-bib-0043] Yang, X., Yang, S., Wang, C., & Kuang, S. (2017). The hypoxia‐inducible factors HIF1alpha and HIF2alpha are dispensable for embryonic muscle development but essential for postnatal muscle regeneration. Journal of Biological Chemistry, 292(14), 5981–5991. 10.1074/jbc.M116.756312 PMC5392588 28232488

[acel13448-bib-0044] Yoon, B. R., Oh, Y. J., Kang, S. W., Lee, E. B., & Lee, W. W. (2018). Role of SLC7A5 in metabolic reprogramming of human monocyte/macrophage immune responses. Frontiers in Immunology, 9, 53. 10.3389/fimmu.2018.00053 29422900 PMC5788887

[acel13448-bib-0045] Zhang, X., Trevino, M. B., Wang, M., Gardell, S. J., Ayala, J. E., Han, X., Kelly, D. P., Goodpaster, B. H., Vega, R. B., & Coen, P. M. (2018). Impaired mitochondrial energetics characterize poor early recovery of muscle mass following hind limb unloading in old mice. Journals of Gerontology. Series A, Biological Sciences and Medical Sciences, 73(10), 1313–1322. 10.1093/gerona/gly051 PMC6132115 29562317

