# Supplementary figures and images for: Disrupted macrophage metabolic reprogramming in aged soleus muscle during early recovery following disuse atrophy
Source: Aging Cell. 2021 Aug 8;20(9):e13448. doi: 10.1111/acel.13448 (PMC8441489; doi:10.1111/acel.13448)

a.

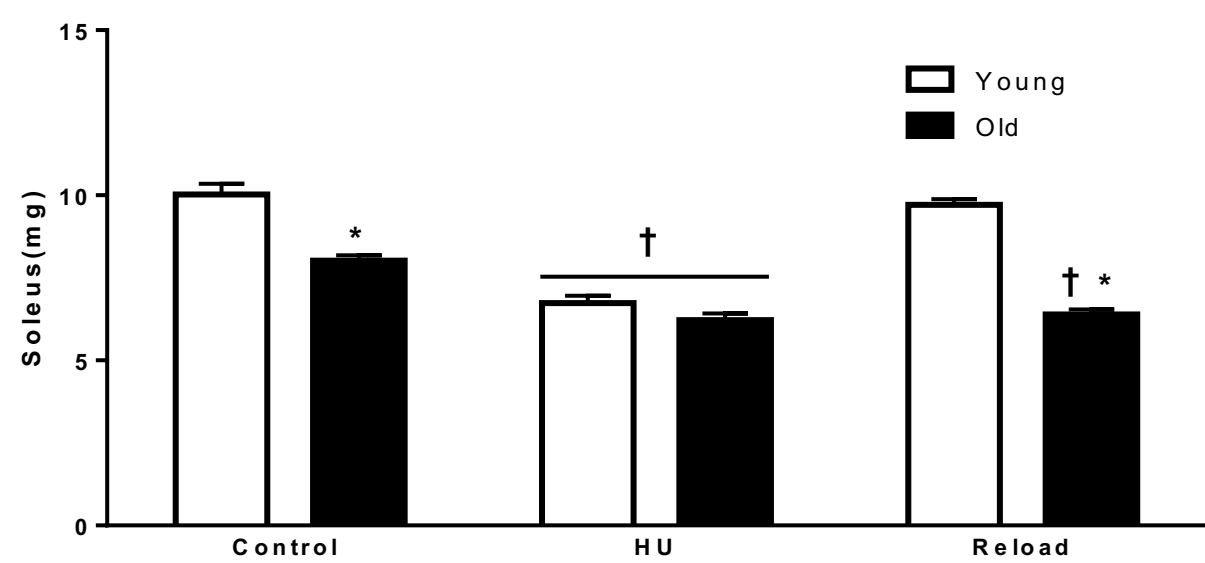

b.

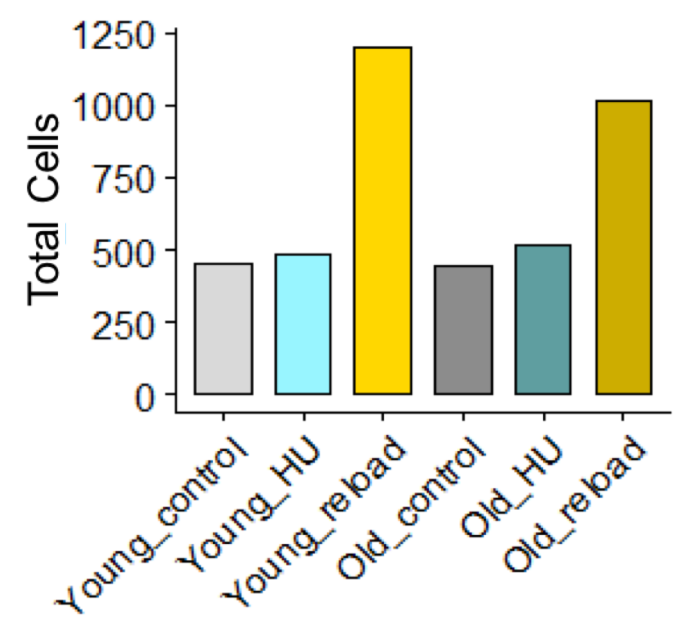

c.

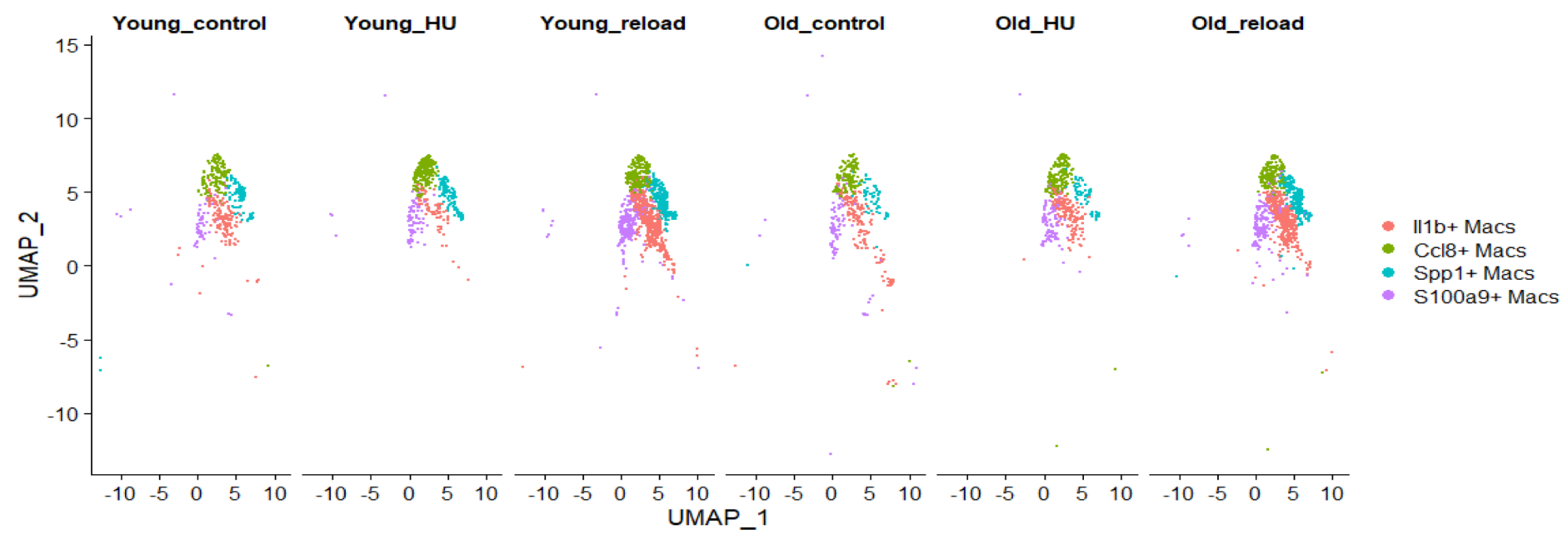

d.

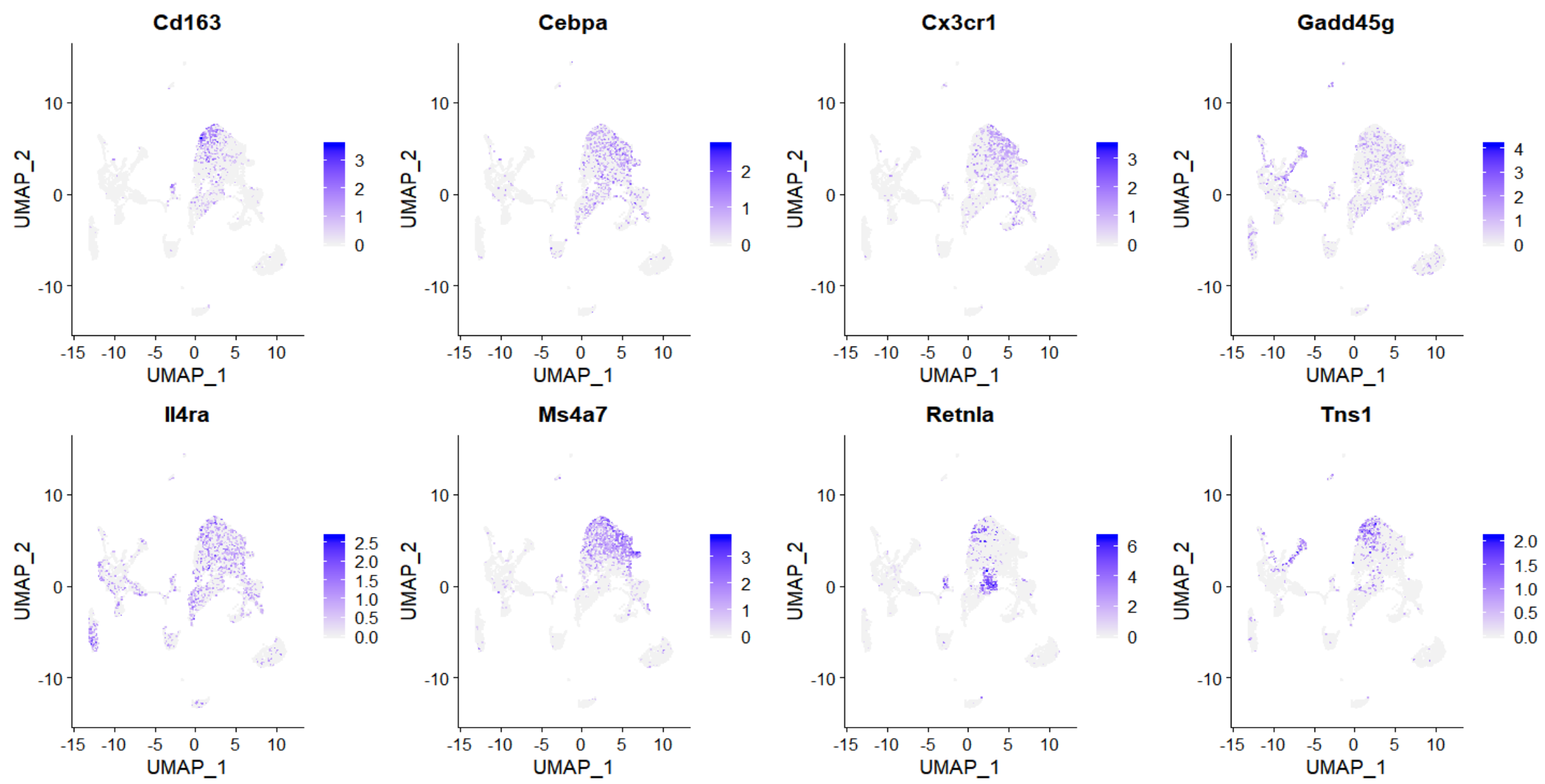

Supplement: Supplementary file 1 — Fig S1 [file ACEL-20-e13448-s001.pdf]

a.

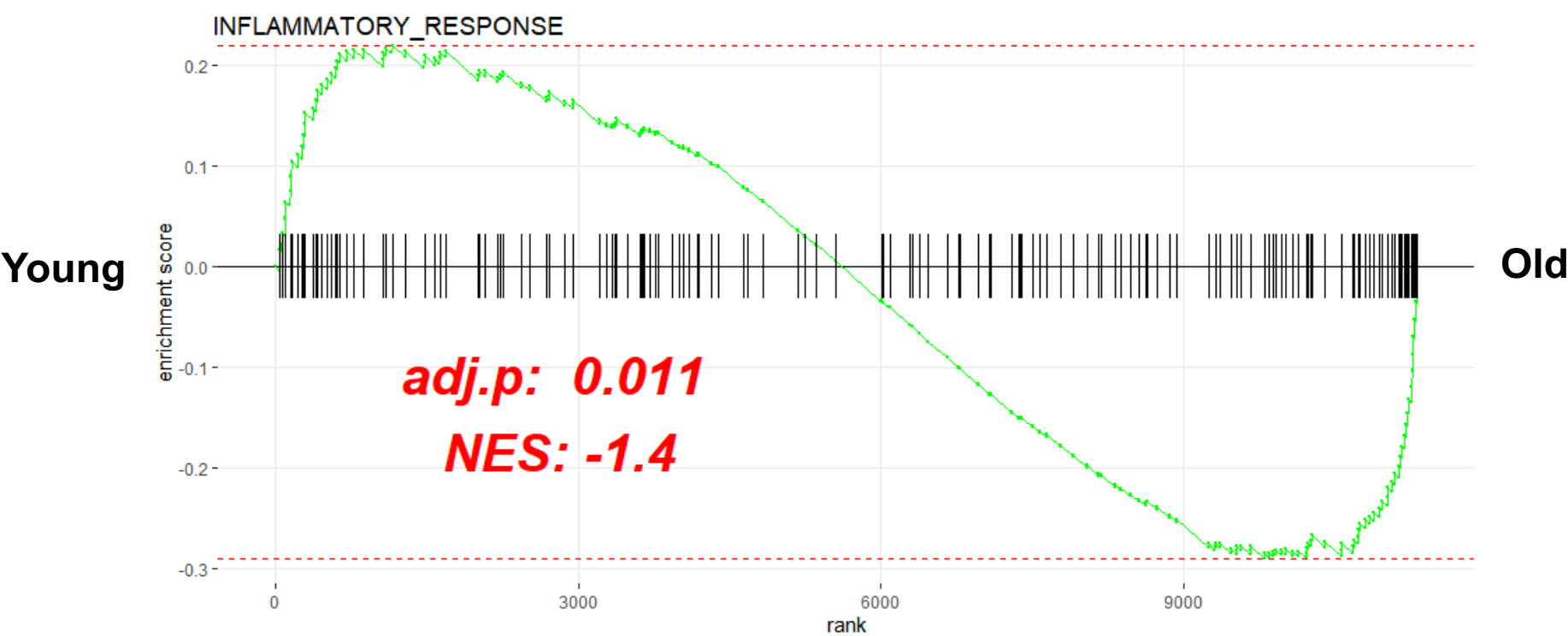

b.

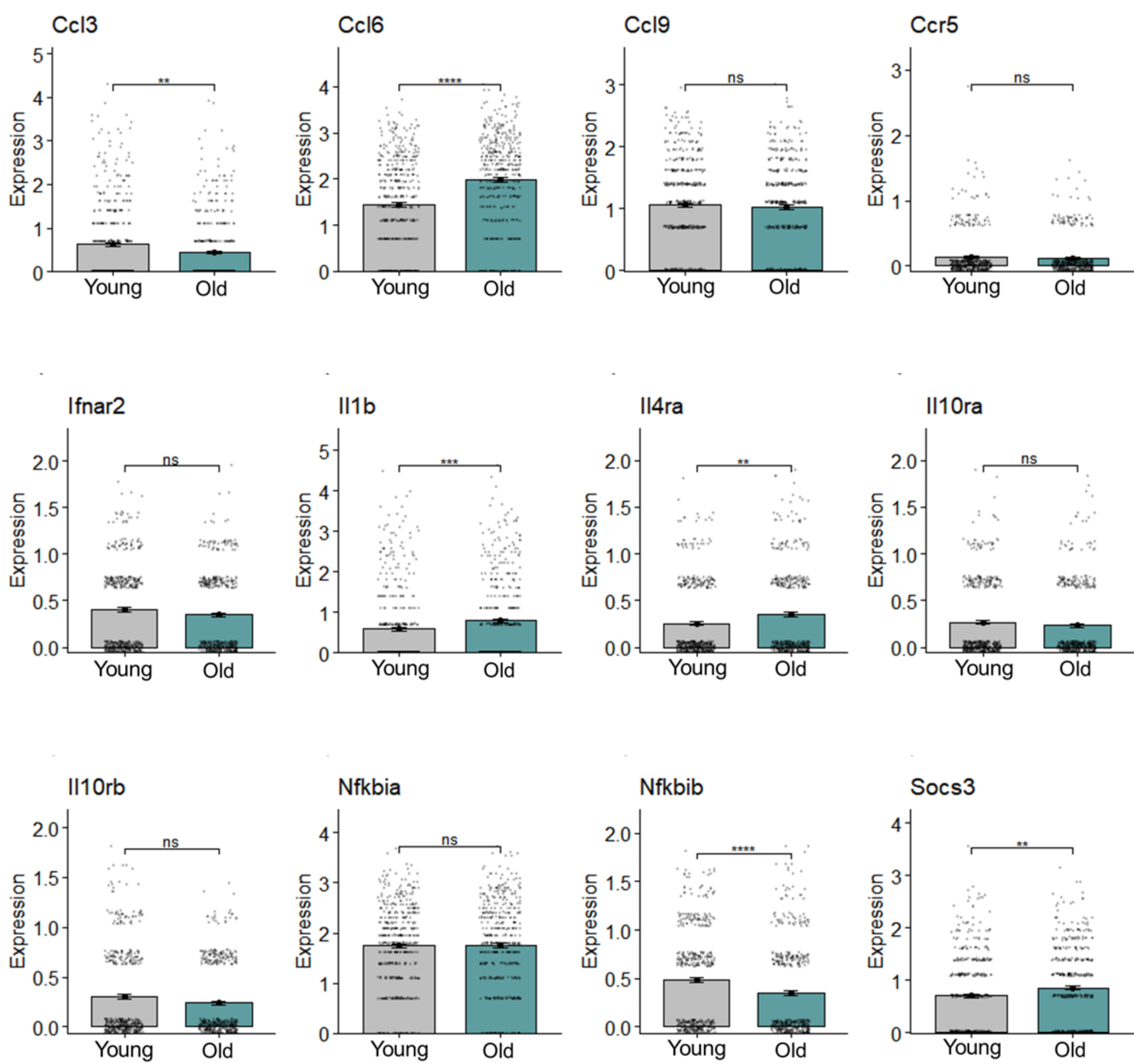

Supplement: Supplementary file 2 — Fig S2 [file ACEL-20-e13448-s004.pdf]

a.

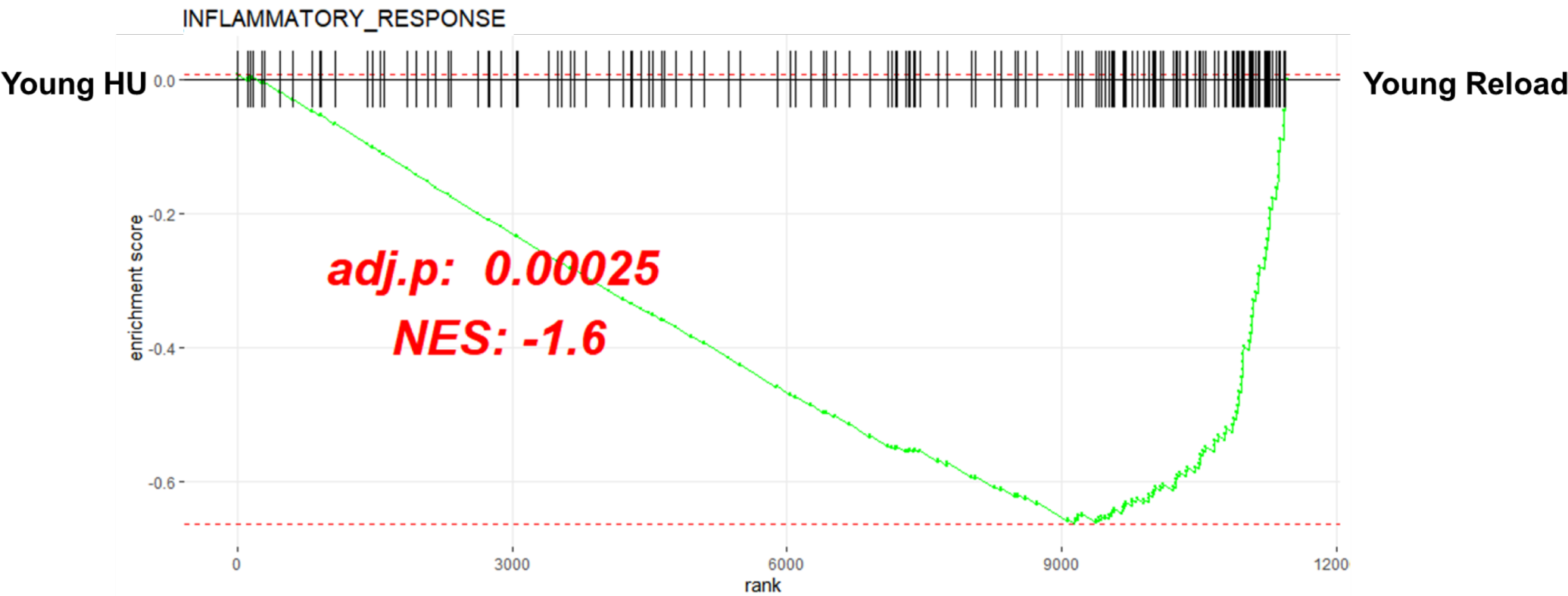

b.

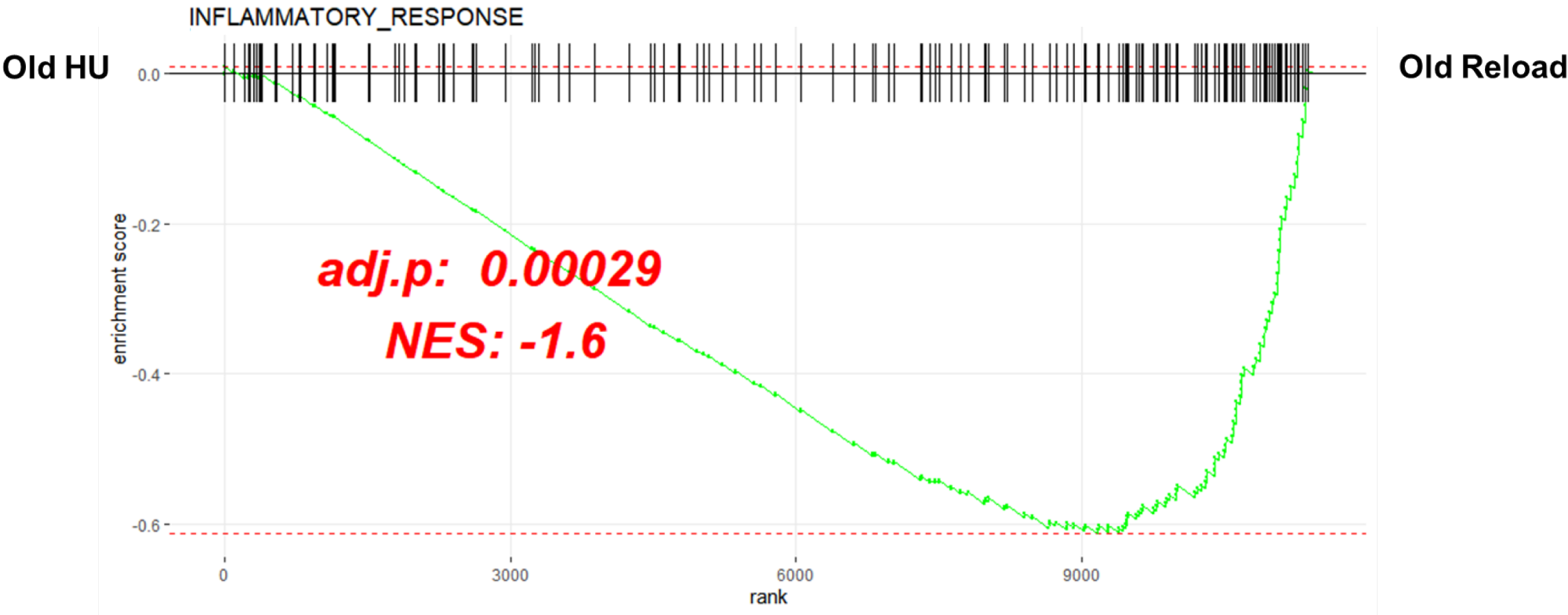

c.

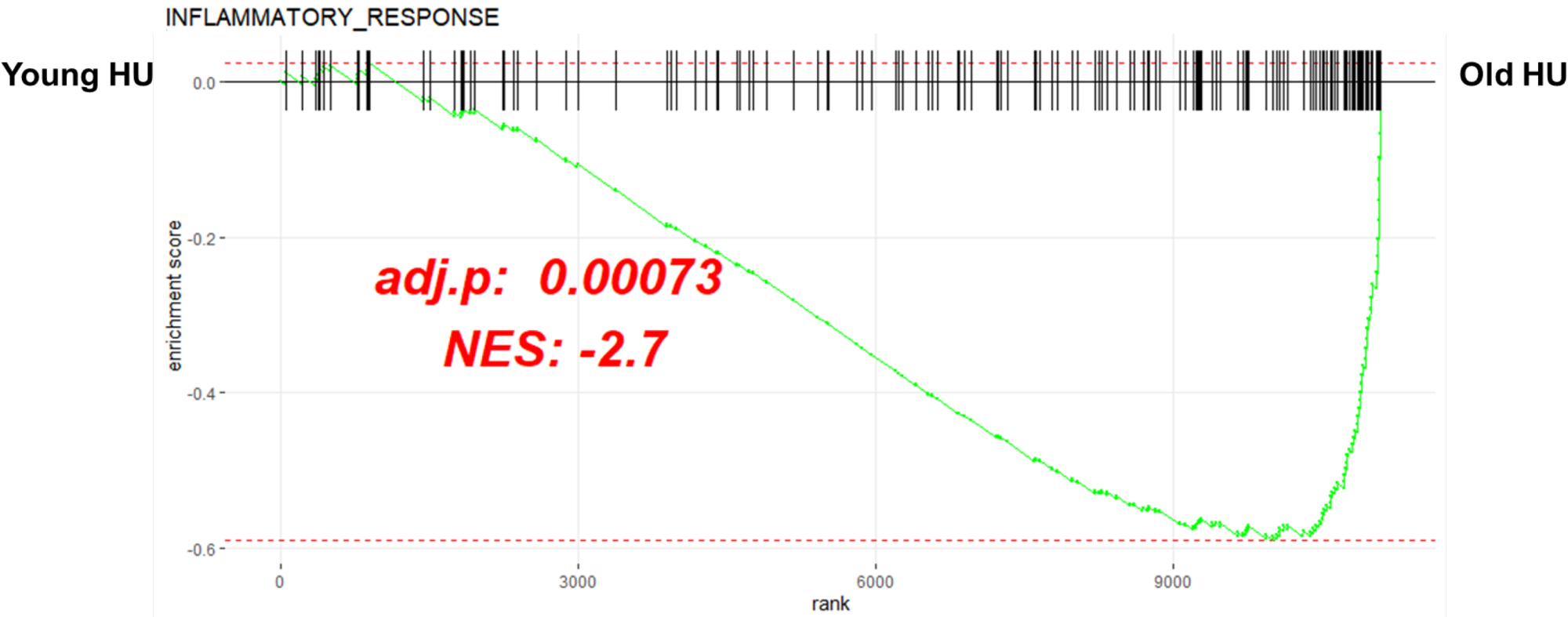

Supplement: Supplementary file 3 — Fig S3 [file ACEL-20-e13448-s002.pdf]

a.

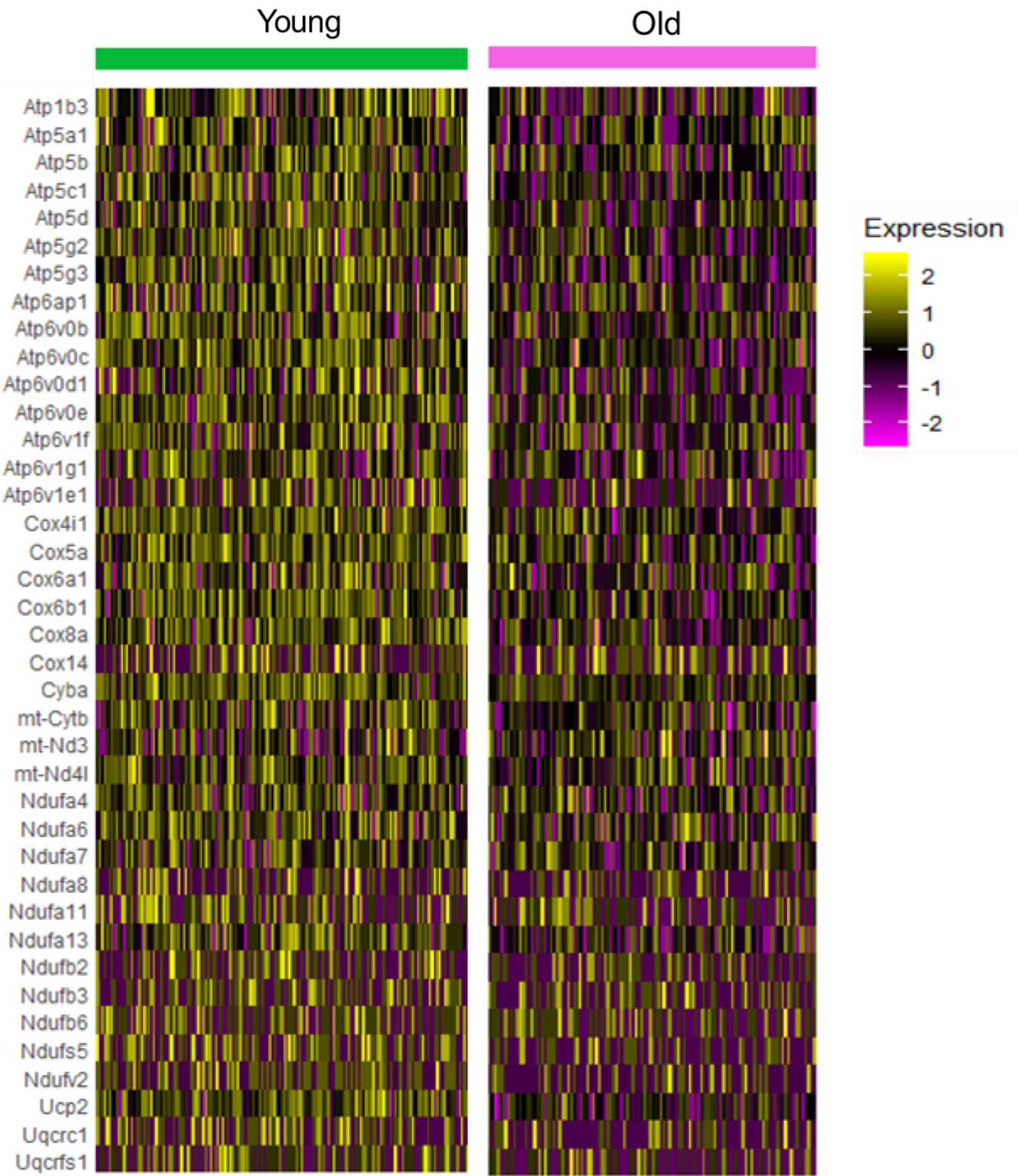

b.

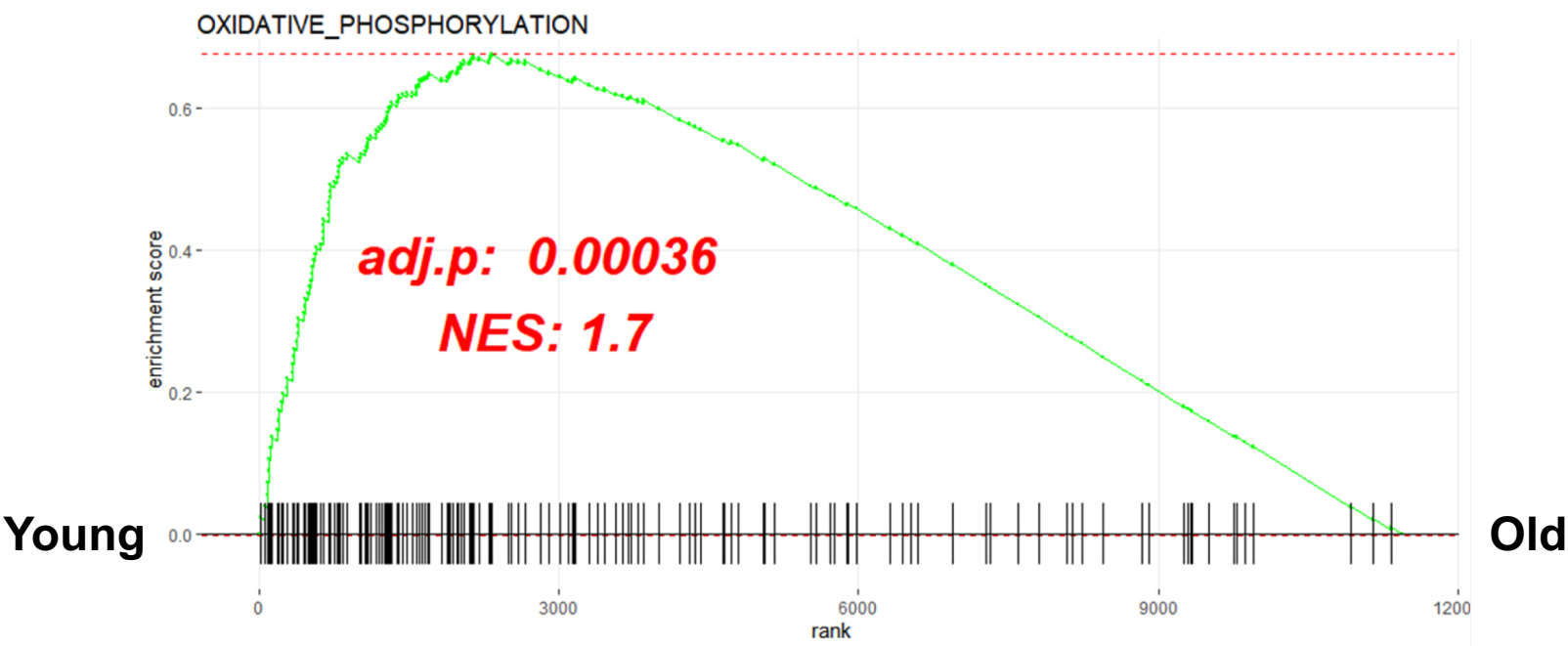

Supplement: Supplementary file 4 — Fig S4 [file ACEL-20-e13448-s003.pdf]
